# Novel 3-(pyrazol-4-yl)-2-(1*H*-indole-3-carbonyl)acrylonitrile derivatives induce intrinsic and extrinsic apoptotic death mediated P53 in HCT116 colon carcinoma

**DOI:** 10.1038/s41598-023-48494-7

**Published:** 2023-12-15

**Authors:** Magda F. Mohamed, Nada S. Ibrahim, Amna A. Saddiq, Ismail A. Abdelhamid

**Affiliations:** 1 Department of Chemistry, College of Science and Arts at Khaulis, University of Jeddah, Jeddah, Saudi Arabia; 2 Department of Chemistry (Biochemistry Branch), Faculty of Science, Cairo University, Giza, Egypt; 3 Department of Biology, College of Science and Arts at Khaulis, University of Jeddah, Jeddah, Saudi Arabia; 4 Chemistry Department, Faculty of Science, Cairo University, Giza, Egypt

**Keywords:** Molecular modelling, Cancer

## Abstract

A novel series of α-cyano indolylchalcones was prepared, and their chemical structures were confirmed based on the different spectral data. Among them, compound **7f** was observed to be the most effective bioactive chalcone with distinguished potency and selectivity against colorectal carcinoma (HCT_116_) with IC_50_ value (6.76 µg/mL) relative to the positive control (5 FU) (77.15 µg/mL). In a preliminary action study, the acrylonitrile chalcone **7f** was found to enhance apoptotic action via different mechanisms like inhibition of some anti-apoptotic protein expression, regulation of some apoptotic proteins, production of caspases, and cell cycle arrest. All mechanisms suggested that compound **7f** could act as a professional chemotherapeutic agent. Also, a molecular docking study was achieved on some selected proteins implicated in cancer (Caspase 9, XIAP, P53 mutant Y220C, and MDM2) which showed variable interactions with compound **7f** with good Gibbs free energy scores.

## Introduction

Cancer cells are defined by variable metabolic changes or cellular reprogramming which allow them to meet their cellular energy needs for growth and metastasis^1^. These different metabolic and bioenergetics pathways enable it to acquire specific features making cancer cells able to survive in conditions with limited resources, based on harnessing other nutrients. If the genetic disorders included in cancer cells and helped it to reprogram itself to resist cell death were identified, it could help in the discovery of novel therapeutic drugs that may improve the outcomes of the clinical studies. One of these genetic disorders is a mutations in TP53 a tumour suppressor gene, confer tumour cells with improved self-renew that helps cancer cells to overcome and compete the apoptosis process^2^. In 2020, the most common cause of cancer related deaths is lung cancer and then colon and rectal cancer^3^. So, it is necessary to intensify our research for synthesizing novel anticancer drugs. It was reported that the indole structure has emerged as one of the most important synthetic sources for therapeutic drugs^4–6^. It has been reported that indole-containing compounds possess a wide range of bioactivities such as antimicrobial^7^, anticancer^8–10^ antihypertensive^11^ antimalarial^12^, analgesic^13^, antidiabetic^14^, anti-inflammatory^15–17^, and anti-HIV activities^18^. Fan Zhang et al., reported the promising anti-tumour activity of 2-amino-3-cyano-6-(1*H*-indol-3-yl)-4-phenylpyridine derivatives against four human cancer cell lines (H460, A549, HT-29 and SMMC-7721)^19^. Additionally, the pyrazole moiety is a potent pharmacological scaffold and has a variety of biological properties, including anti-microbial^20, 21^, anti-inflammatory^22–24^, anti-tubercular^25, 26^, anticancer activity^27–30^, antidepressant activity^31^, and anti-fungal activity^32^. Moreover, the compounds bearing 1,3-diaryl-2-propen-1-one (chalcones) are distinguished secondary metabolites that are reported to exhibit various bioactivities such as antiplatelet^33^, antiviral^34^, anti-inflammatory^35–37^, antimalarial^38^, antibacterial^39, 40^, analgesic^41^, anticancer^40, 42–44^, and antioxidant^36, 42^. The presence of a reactive *a*,*ß*-unsaturated enone group in chalcones is assumed to be responsible for their bioactivity, thus several articles on new chalcone derivatives have been reported with structural modifications around the basic enone unit. The creation of carbon–carbon bonds is the most significant process in the synthesis of organic compounds^43, 45–53^. In a study, novel chalcone derivative were shown to exert anticancer activities in MCF-7 breast carcinoma^54^. Similarly, in another study, the cytotoxic effect of a number of synthesized chalcone derivatives were tested in five human cancer cell lines (A549, MCF-7, HL-60, SW480, and SMMC-7721,), and some of the chalcones showed significant cytotoxic effects by triggering cell cycle arrest and apoptosis^55^. Coskun et al.^56^ studied the cytotoxic and apoptotic effects of a series of chalcones in A549, MCF-7, and PC-3 cells and the results showed that they triggered apoptosis through caspase dependent pathway. The Knoevenagel condensation is one of the most common reactions that lead to the synthesis of olefins with biological importance^57–60^. It was assumed that the molecular hybrid combination of indole, pyrazole, and chalcone moieties together in a molecular framework would produce molecules with high bioactivity. Motivated by the above-mentioned facts, herein we report the synthesis and anticancer activity of α-cyano indolylchalcones incorporating 1-phenyl-3-arylpyrazole.

## Results

### Chemistry

**Figure 1:**
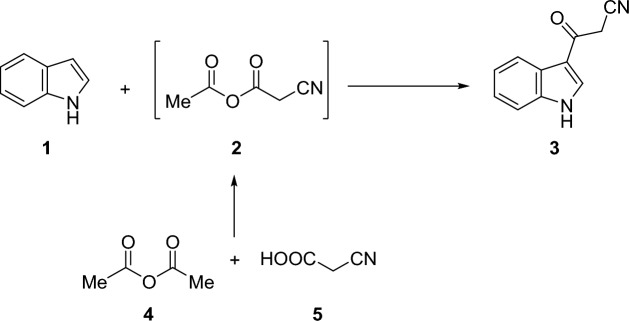
Synthesis of the 3-(cyanoacetyl)indole precursor **3.**

Initially, the precursor required for the synthesis of our targets viz. 3-(1*H*-indol-3-yl)-3-oxopropanenitrile **3** was prepared from the reaction of indole **1** with the cyanoacetylating intermediate **2** that resulted from a preheated mixture of acetic anhydride **4** and 2-cyanoacetic acid **5** (Fig. 1).

**Figure 2:**
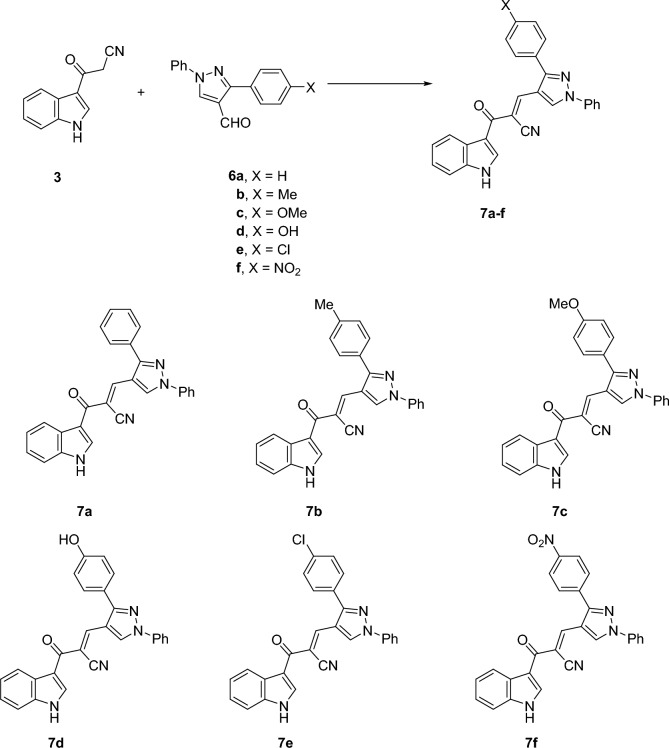
Synthesis of *α*-cyano indolylchalcones incorporating 1-phenyl-3-arylpyrazole moiety **7a–f.**

Subsequently, a new library of *α*-cyano indolylchalcones **7a–f** incorporating 1-phenyl-3-arylpyrazole moiety was prepared in excellent yields via the Knoevenagel condensation reaction of equimolar amounts of 3-(cyanoacetyl)indole **3** with pyrazole-aldehydes **6a–f** in ethanol in the presence of piperidine as a catalyst (Fig. 2). The constitutions of the novel compounds were confirmed based on the different spectral tools. Thus, ^1^H NMR of **7a** as a representative example indicated three singlets at 8.11, 8.52, and 9.25 ppm corresponding to vinyl-H, indole-H2, and pyrazole-H5, respectively. The broad singlet at 12.22 is assigned to NH. Besides, it showed mutiplets of aromatic protons in the aria of 7.24–8.52 ppm. Furthermore, the structure was proved based on ^13^C NMR that indicated the presence of 23 signals corresponding to 23 different carbons.

### Antiproliferative effect of *α*-cyano indolylchalcones

**Figure 3:**
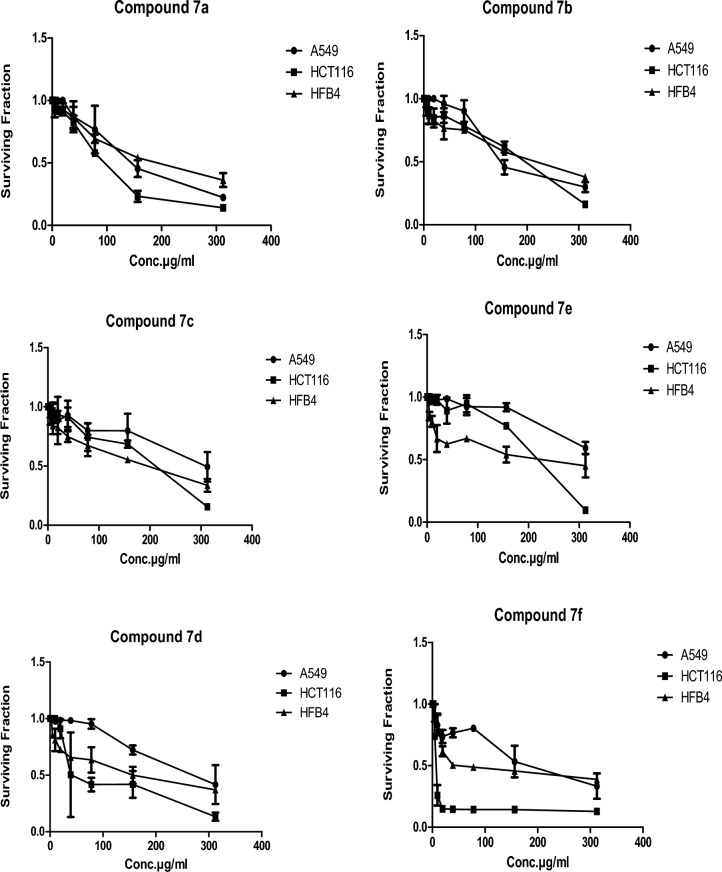
Cytotoxic effect of the synthesized compounds against the tested cancer cell lines (A549 and HCT116) and normal melanocytes HFB4 cells. The graphs were plotted with standard deviation (SD) using the Prism software program (Graph Pad software incorporated, version 3).

The prepared compounds **7a–f** were screened against two cancer cell lines, lung carcinoma (A549), and colon cancer (HCT116). Also, the compounds were tested against the normal human skin melanocyte cell line (HFB4) for the sake of comparison. As indicated in Table 1 and Fig. 3, the cytotoxic effect of our synthesized series against HCT116 was better than the A549 cell line. The IC_50_ values ranged from 6.76 to 202.08 µg/mL for HCT116, while they ranged from 193.93 to 1,014.00 µg/mL for A549. Concerning the HCT116 cell line, it was found that compound **7f** was the most promising compound (IC_50_ = 6.76 µg/mL) compared to 5 fluorouracil (positive control) (IC_50_ = 77.15 µg/mL). Compound **7d** exerted a good cytotoxic effect (IC_50_ = 43 µg/mL) relative to the positive control, while the IC_50_ value for compound **7a** was 93.1 µg/mL which means moderate activity. The remaining compounds **7b**, **7c**, and **7e** showed weak cytotoxic activity (IC_50_ = 187.81, 202.08, and 195.41 µg/mL, respectively). Concerning A549, all the tested compounds showed lower IC_50_ values than the positive control (IC_50_ = 371.36µg/mL) except for compound **7e** which exerted a very weak effect (Table 1). Among them, compound **7f** showed the lowest IC_50_ value (193.93µg/mL) relative to 5fluorouracil. While, compounds **7a**, **7b**, **7c**and **7d** showed comparable IC_50_ values (208.58, 238.14, 274.60, and 269 µg/mL, respectively). From the results of the cytotoxicity against normal HFB4 cells (Table 1), it was noticed that compound **7f** was the most selective toward HCT116 (SI = 8.4), (Selectivity index (SI) = IC_50_ in normal cells/IC_50_ in tumor cells).

**Table 1:** IC_50_ values for the prepared compounds against the selected cell lines (A549, HCT116, and HFB4), 5-Fluorouracil was used as a positive control.

Compounds	IC_50_ values (µg/mL)		
	A549	HCT116	HFB4
**7a**	208.58	93.1	184
**7b**	238.14	187.81	228.8
**7c**	274.60	202.08	164.5
**7d**	269	43	156.25
**7e**	1,014.00	195.41	192
**7f**	193.93	6.76	56.7
Positive control (5 FU)	371.36	77.15	222.67

#### Structure–activity relationship

**Figure 4:**
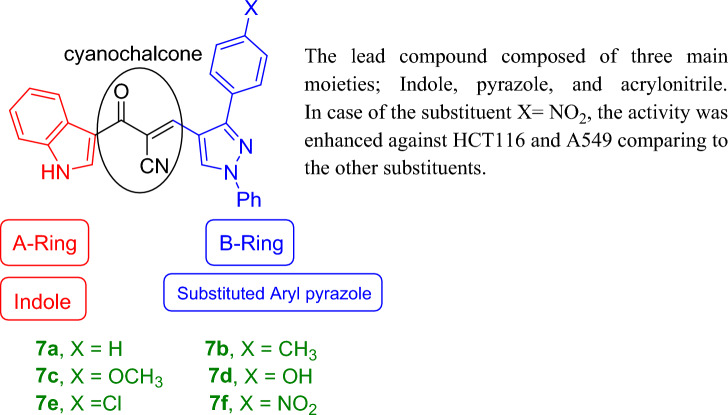
Structure–activity relationship of the synthesized compounds **7a–f**.

The structure–activity relationship of the prepared compounds toward HCT116 can be regarded in view of the substitution on the arly group at position-3 of the pyrazole ring. In the case of X = NO_2_ as in compound **7f** (Fig. 4), the cytotoxic activity and selectivity increased toward HCT116 as compared to the other compounds. It was noticed that compound **7d** (X = OH) had better anticancer activity than the positive control and was selective toward HCT116 cells. When X = H as in compound **7a**, the activity was found to be moderate. For compounds **7b**, **7c**, and **7e** (X = CH_3_, OCH_3_, and Cl) respectively, the activity was lower than that of the standard. Concerning A549, the activity of all compounds of the series except compound **7e** (X = Cl) was better than the positive control and the best of them was compound **7f** (X = NO_2_). But the selectivity of the prepared compounds deteriorated against A549.

### Gene expression analysis

**Figure 5:**
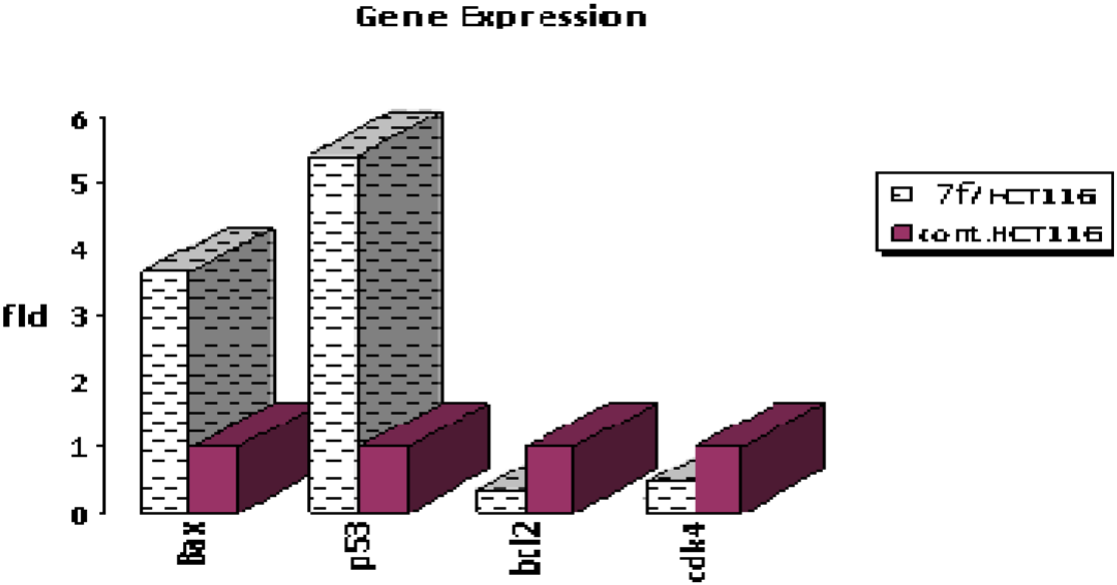
The schematic diagram outlined expression values of tested four genes (*Bax, p53, Bcl2*, and *CDK4*) for both control and cells treated with chalcone **7f**.

In this current research, relative gene expression related to apoptosis should be studied well because they were very interesting to understand the effect of our selected compound on this important biological process. HCT_116_ cells were treated with chalcone **7f** (6.76µg/mL**)** for 48 h, and then subjected to qPCR analysis regarding control samples. The effect of chalcone **7f** on the expression level of (*Bax, p53, Bcl2,* and *CDK4*) genes was assessed using the qPCR technique. As illustrated in Table 2 and Fig. 5, the results indicated that mRNA expression values of tested apoptotic (*Bax* and *p53*) and anti-apoptotic (*Bcl2* and *CDK4*) genes outlined promising and interesting effects regarding negative control. As indicated in this schematic diagram, the expression of apoptotic genes (*Bax* and *P53*) was enhanced by the effect of chalcone **7f** to reach (3.662, and 5.435) respectively, relative to the control. Also, from the resulting plot, the anti-apoptotic genes (*bcl2* and *CDK4*) were down-regulated effectively to values (0.351 and 0.454) respectively, by 6.76µg/mL of chalcone **7f** in comparison with negative control.

**Table 2:** RT-PCR data illustrated expression folding of (*Bax, p53, Bcl2,* and *CDK4*) genes for tested chalcone relative to untreated HCT_116_ cells.

Samples	RT-PCR fold change			
	*Bax*	*p53*	*bcl2*	*CDK4*
7f/HCT116	3.662	5.435	0.351	0.454
cont.HCT116	1	1	1	1

### Elisa assay

#### Effect of chalcone 7f on *caspase 3* production in HCT116

**Figure 6:**
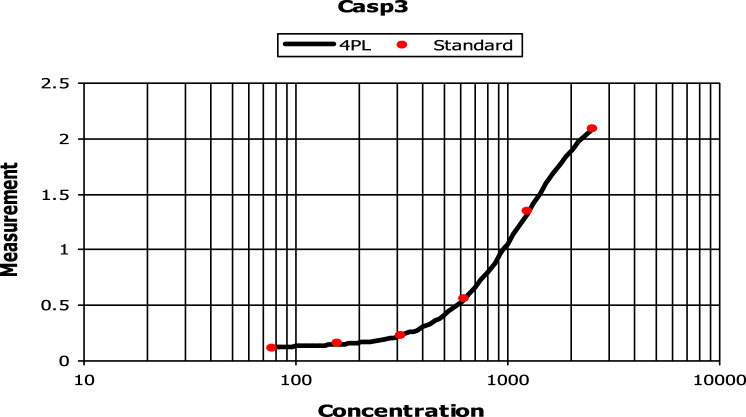
The effect of various concentrations of **7f** on *caspase 3* production in colorectal carcinoma. HCT116 cells were incubated in the absence (control) or presence of different concentrations of **7f**, after which the *caspase 3* protein level was measured by ELISA.

**Figure 7:**
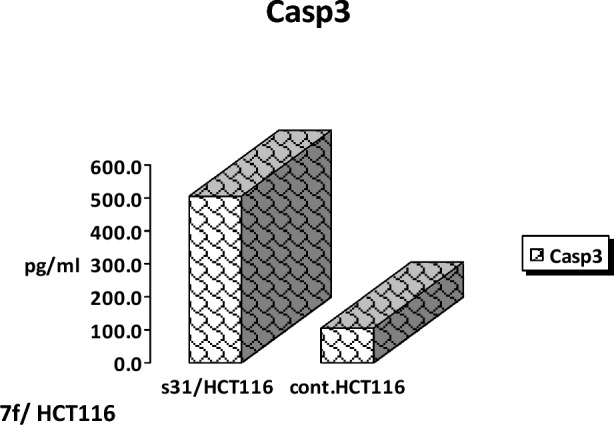
The effect of chalcone **7f** on *caspase 3* production in colorectal carcinoma. HCT116 cells were incubated in the absence and presence of **7f**, after which the *caspase 3* protein level was measured by ELISA.

Apoptosis is known and identified by specific characterizations in which the activation of *caspases* plays an important role. In apoptotic pathways, mechanisms of (activation/inactivation) of these proteins are not fully understood. The significance of understanding the mechanistic machinery that controls programmed cell death, being initiated by various chemotherapeutic agents is very critical and attracts the attention of researchers in this area. In this assay, cells were incubated in the absence or presence of **7f** with varying concentrations (39, 78, 156, 313, 625, 1250, and 2500 ng/ml), inducing a concentration-dependent increase in *caspase3* production in HCT116. The maximal increase in *caspase 3* production occurred with **7f** concentrations between 1250 ng/ml and 2500 ng/ml as illustrated in Fig. 6. A concentration of **7f** was used at its IC_50_ value and compared directly to the control. Incubation of **7f** with HCT116 resulted in high production of 504.1 pg/ml of *caspase 3* protein. However, there was only 103.5 pg/ml induced by control cells. It was interesting to note that chalcone **7f** stimulated the production of *caspase 3* protein in HCT116 cells 4.8-fold compared to control as outlined in Fig. 7.

### Effect of chalcone 7f on *caspase 8* production in HCT116

**Figure 8:**
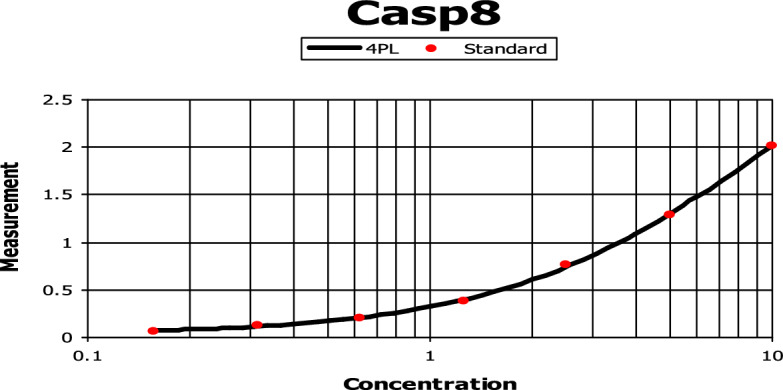
The effect of various concentrations of **7f** on *caspase 8* production in colorectal carcinoma. HCT116 cells were incubated in the absence (control) or presence of different concentrations of **7f**, after which the *caspase 8* protein level was measured by ELISA.

**Figure 9:**
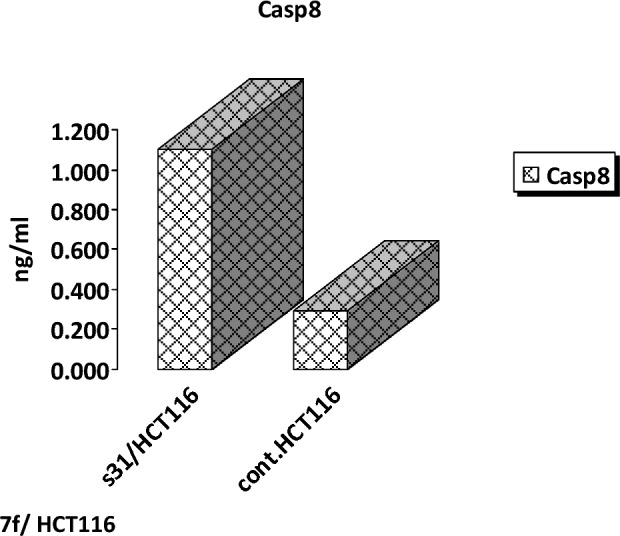
The effect of chalcone **7f** on *caspase 8* production in colorectal carcinoma. HCT116 cells were incubated in the absence and presence of **7f**, after which the *caspase 8* protein level was measured by ELISA.

The production of *caspase 8* was determined using various concentrations of **7f** in a concertation-dependent manner. There was a gradual increase in the *caspase 8* production level using the following concentrations (0.156, 0.312, 0.625, 1.25, 2.5, 5, and 10 ng/ml). The maximal increase in *caspase 8* production was achieved by using **7f** concentrations between 5 and 10 ng/ml (Fig. 8). HCT116 cells treated with **7f** resulted in a 3.788-fold increase in *caspase 8* production level. It achieved 1.104 ng/ml of *caspase 8* compared to the untreated HCT116 cell line which was able to produce only 0.2914 ng/ml of *caspase 8* (Fig. 9).

### Effect of 7f compound on *caspase 9* production in HCT116

**Figure 10:**
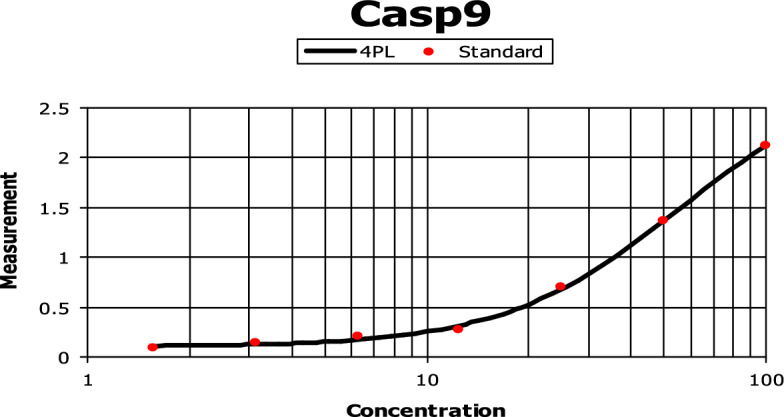
The effect of various concentrations of **7f** on *caspase 9* production in colorectal carcinoma. HCT116 cells were incubated in the absence and presence of different concentrations of **7f**, after which the *caspase 9* protein level was measured by ELISA.

**Figure 11:**
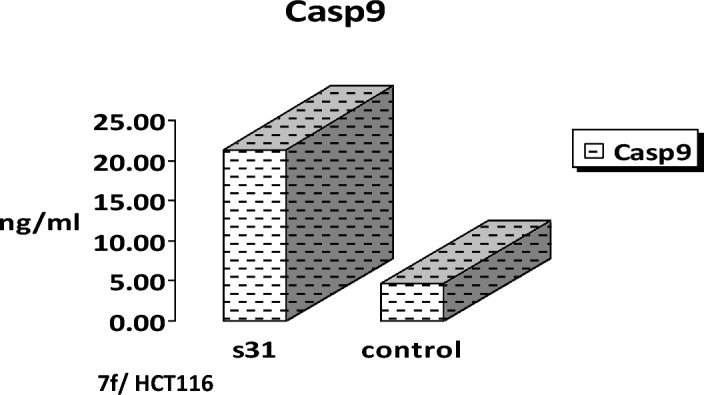
The effect of chalcone **7f** on *caspase 9* production in colorectal carcinoma. HCT116 cells were incubated in the absence and presence of **7f**, after which, the *caspase 9* protein level was measured by ELISA.

The compound **7f** induced *caspase 9* production in a dose–response approach. This was achieved by using the following concentrations of chalcone **7f** (1.6, 3.1, 6.3, 12.5, 25, 50, 100 ng/ml). There was a continuing rise in the *caspase 9* production level by increasing the concentrations regularly. A concentration between 50 and 100 ng/ml induced a maximum *caspase 9* production as shown in Fig. 10. Compound **7f** was able to stimulate the production of *caspase 9* by inducing a 21.43 ng/ml of *caspase 9*. This showed about a 4.641-fold increase compared to control cells (Fig. 11).

### Flow cytometer analysis

**Figure 12:**
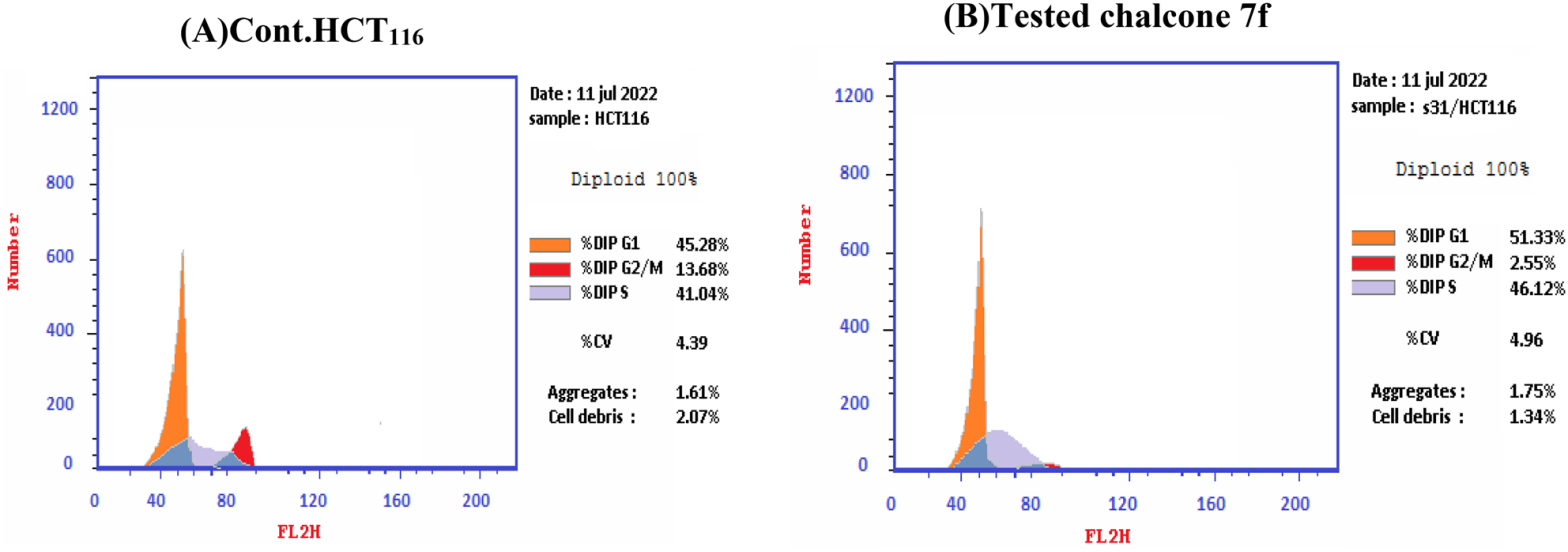
Cell cycle- analysis of chalcone**7f** in HCT116 cells. (A) control HCT 116 cell line; (B) HCT116 cells treated with chalcone **7f**.

**Figure 13:**
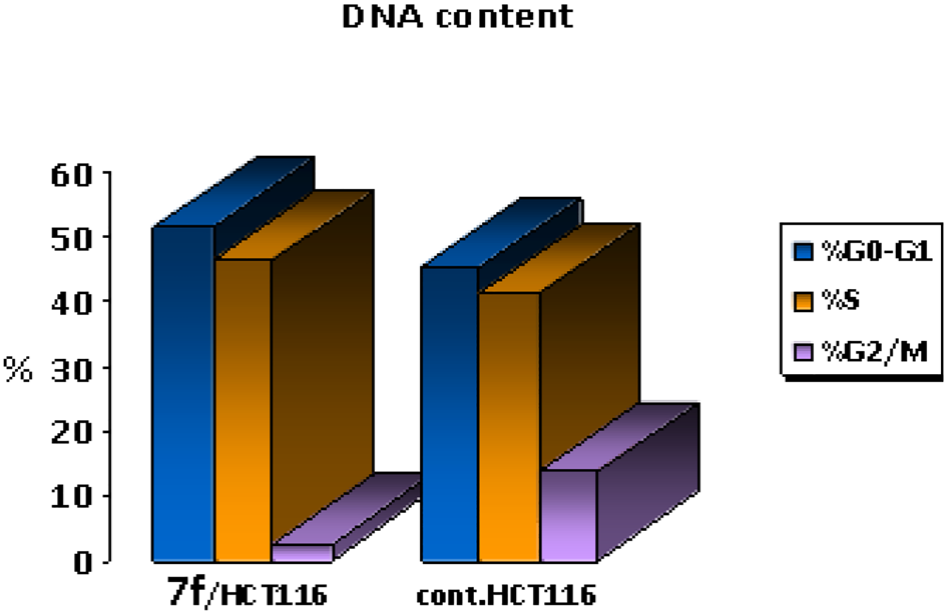
DNA contents in each cycle of tested chalcone **7f** and control HCT _116_ cell line.

**Figure 14:**
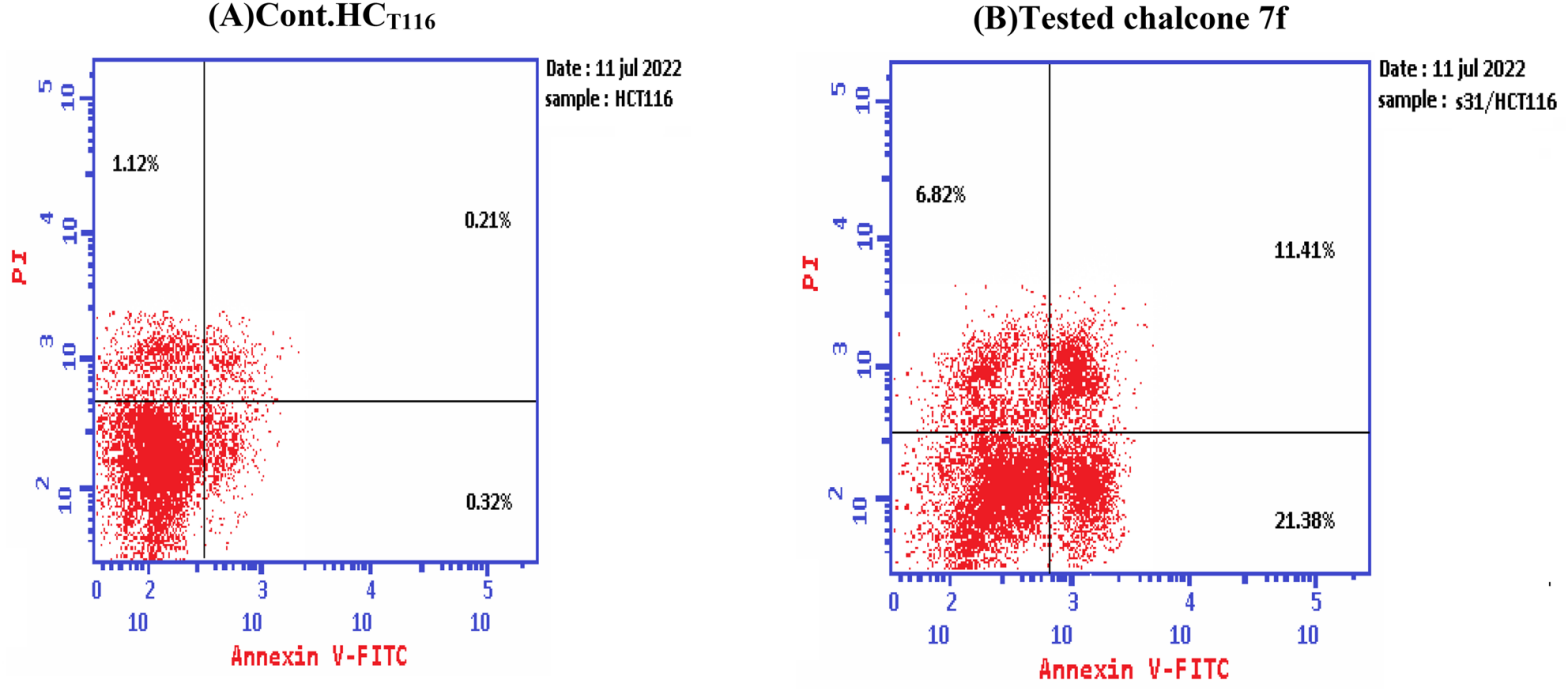
Cell cycle analysis of chalcone **7f** treated HCT116 cells relative to control HCT _116_ cell line.

**Figure 15:**
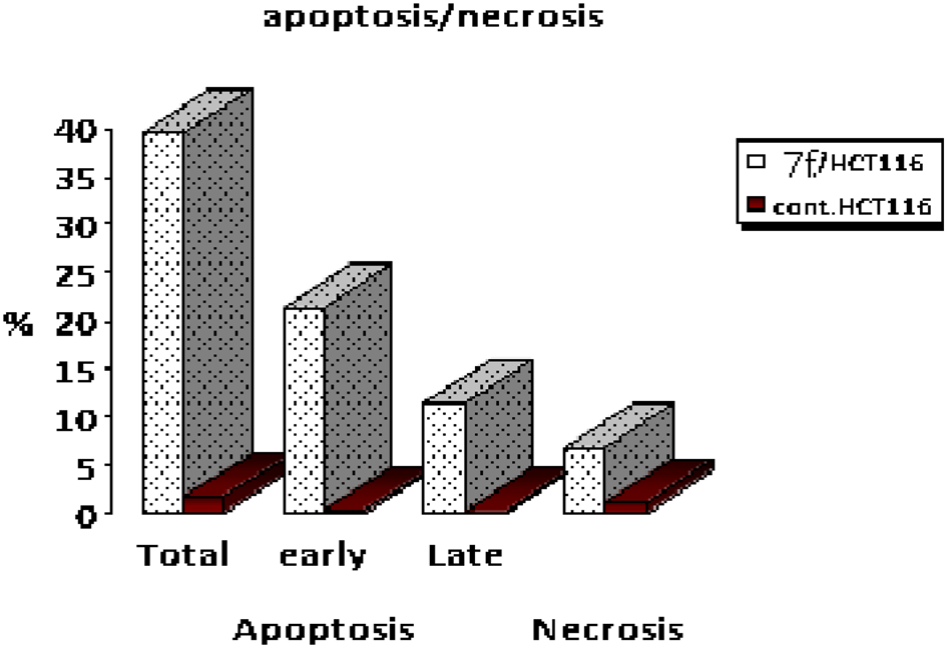
The schematic diagram illustrated the percentages of the total, early, late apoptosis, and necrosis of control and treated sample **7f**.

Chalcone **7f** was selected for more investigations, in attempts to find its molecular action on cancer cells. It illustrated high and very interesting cytotoxicity toward the tested colon cancer cell line (HCT116) as mentioned in the above MTT assay data (Table 1). A flow cytometric report was utilized in this recent paper to investigate the effect of our novel acrylonitrile compound **7f** on the cell cycle, HCT_116_ cells were treated with compound **7f** at a dose of (6.76 µg/mL) for 48 h and subjected to flow cytometric analysis. The data presented in Figs. 12 and 13 obviously indicated that chalcone **7f** caused cell-growth arrest at **G1/S** phase relative to the untreated control HCT_116_ cell line. At 6.76 µg/mL, compound **7f** showed (51.33%) cell accumulation in the G1/S phase at the expense of other phases especially the mitotic phase G2/M phase (2.55%). Also, it was clearly appeared that chalcone **7f** stimulated apoptotic induction of colon cancer cells significantly to reach 21.38% compared to control cells (0.32%) as shown in Figs. 14 and 15.

### DNA fragmentation

**Figure 16:**
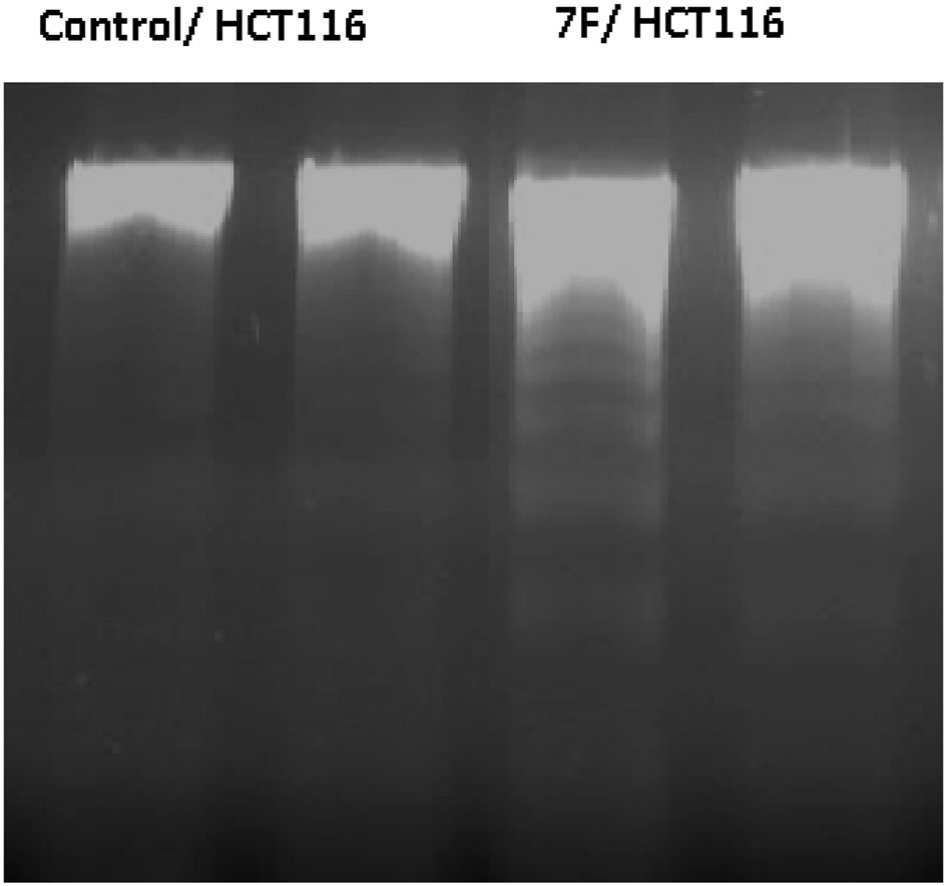
The 2% Agarose gel electrophoresis diagram showed DNA samples of both untreated HCT116 cells and cells treated with 6.76 µg/mL of chalcone 7f. after 48h of treatment.

Both control and treated cells were incubated for 48h. Then cells of both two samples were harvested for DNA fragmentation measurements using a SIGMA DNA fragmentation imaging kit with catalogue number 06432344001. To verify the validity of the DNA of both control and cells treated with 6.76 µg/mL of chalcone 7f, we carried out the loading of all samples on 2% agarose gel containing 1 µg/mL ethidium bromide and visualized by ultraviolet transillumination. As indicated in Fig. 16, a duplicate run for each sample was performed. It was illustrated that chalcone **7f** completely deteriorated the genomic DNA of colorectal carcinoma after 48 h of treatment with respect to the control sample. The finding was promising and surprising and suggested that chalcone **7f** was a promising chemotherapeutic agent.

### Molecular docking study on compound 7f

**Figure 17:**
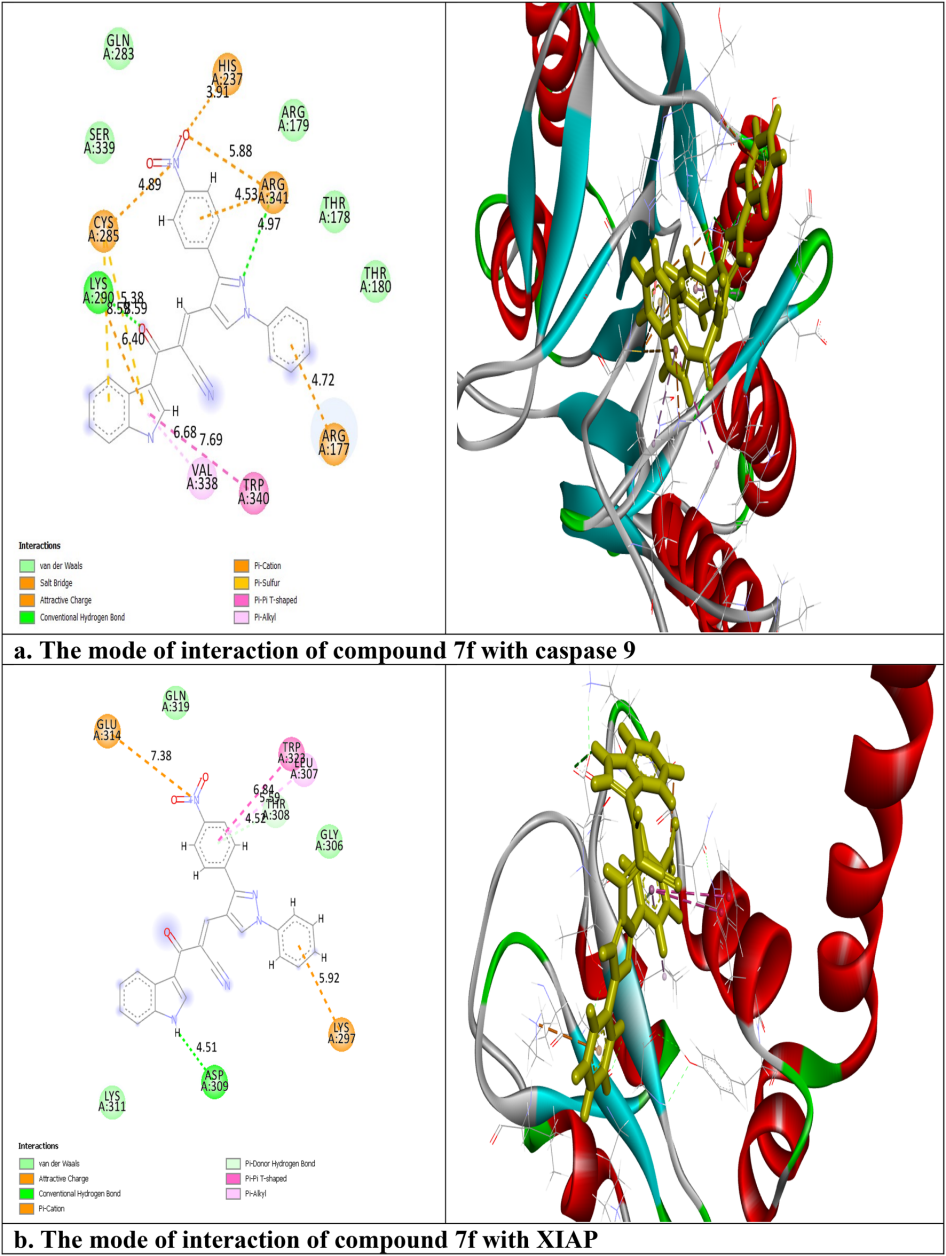

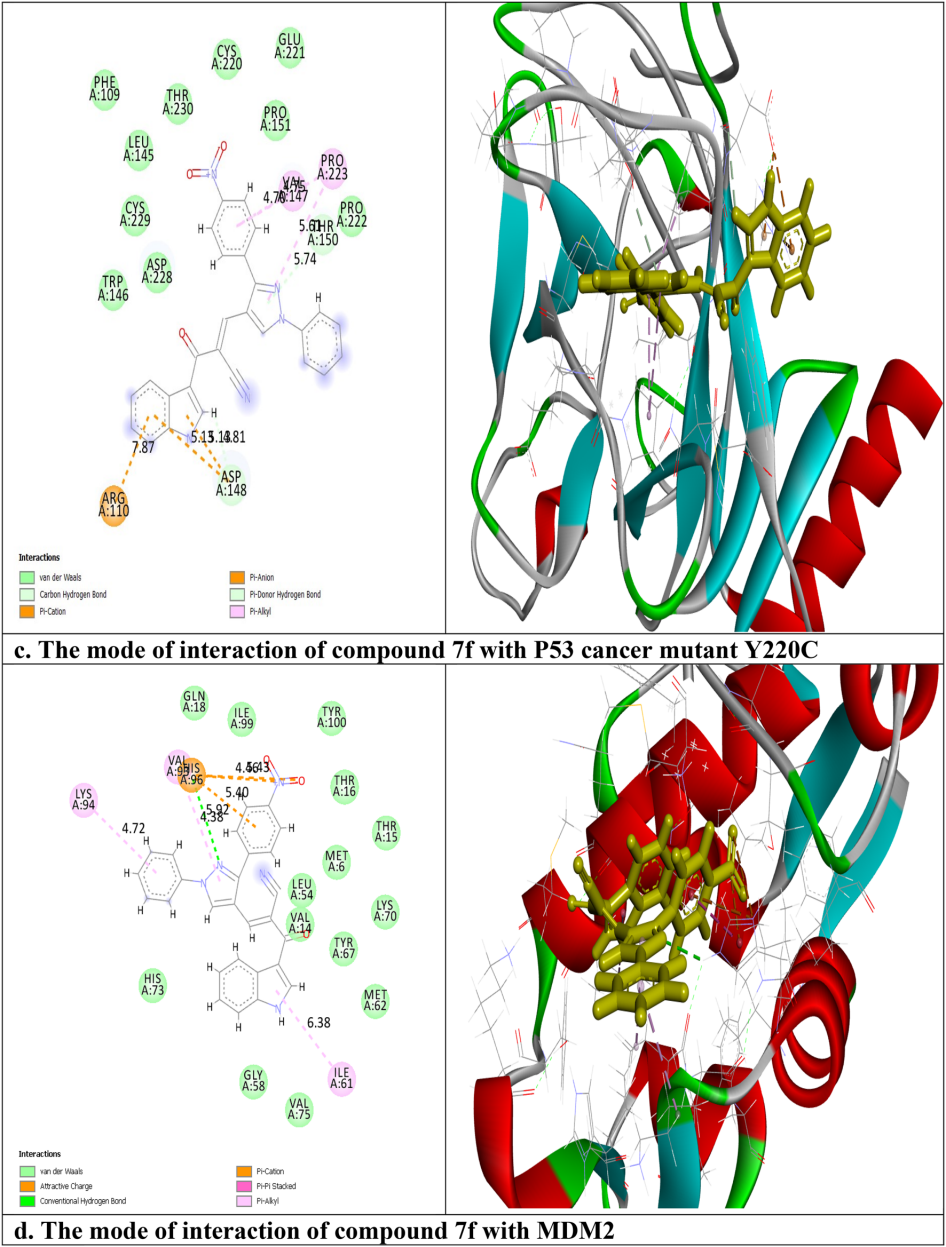
Two-dimensional (2D) and three-dimensional (3D) representation of binding of compound **7f** with the active domain of (**a**) caspase 9, (**b**) XIAP, (**c**) P53 cancer mutant Y220C and (**d**) MDM2 proteins.

The interactions of compound **7f** with the crystal structure of caspase 9, X-linked inhibitor of apoptosis protein (XIAP), P53 cancer mutant Y220C, and Mouse double minute 2 homolog (MDM2) proteins (PDB code: 1jxq, 3eyl, 5o1h and 4wt2, respectively) were examined using molecular Operating Environment and BIOVIA Discovery Studio programs. As shown in Table 3, the RMSD values ≤ 2 (2, 0.86, 0.7, and 2 respectively) which demonstrated the high accuracy of our molecular docking results. Also, it was reported from Table 3 that the binding energies for compound **7f** were − 21.8, − 16.5, − 20.1, and − 24.7 kcal/mol respectively, which were comparable to that of the co-crystallized standard ligands (− 33.7, − 24.9, − 15.8, and − 33.9 kcal/mol respectively). Figure 17a showed that compound **7f** interacted with caspase 9 through 12 interactions, one hydrogen bond between the nitrogen of pyrazole moiety and ARG: 341 with bond distance 4.97 Å, another hydrogen bond between the oxygen of carbonyl group and LYS: 290 with bond distance 5.38 Å, two π-sulfur electrostatic interactions between the indole ring and CYS: 285, six π-cation electrostatic interactions with ARG: 177, ARG: 341, HIS: 237, CYS: 285 and LYS: 290, π–π T-shaped hydrophobic interaction between the indole ring and TRP: 340, and $\pi$- alkyl hydrophobic interaction between the indole ring and VAL: 338. Regarding XIAP, compound **7f** was bound with 6 interactions (Fig. 17b). These interactions were assigned as follows, a hydrogen bond between NH of indole ring and ASP: 309 (4.51 Å), π-donor hydrogen bond between benzene and LEU: 307 (4.52 Å), π–cation electrostatic interaction between phenyl ring and LYS: 297, π–π T-shaped and π-alkyl hydrophobic interactions between the benzene ring and, TRP: 323 and LEU: 307 respectively, and electrostatic interaction between the nitrogen of the nitro group and GLU: 314. While P53 cancer mutant Y220C was activated via 8 interactions. It was shown from Fig. 17c that these interactions included a carbon-hydrogen bond with ASP: 148 with bond distance 3.81 Å, π- donor hydrogen bond between the pyrazole ring and THR: 150 with bond distance 5.74 Å, three π-cation electrostatic interactions with ARG: 110 and ASP: 148 and three π- alkyl hydrophobic interactions with PRO: 223. At last, 7 interactions were seen between MDM2 and compound **7f** (Fig. 17d). Among them, one hydrogen bond between the nitrogen of pyrazole ring and HIS: 96 (5.92 Å), one $\pi$—alkyl hydrophobic interaction between phenyl moiety and LYS: 94, one π-alkyl interaction between pyrazole moiety and VAL: 93, one π-alkyl interaction between indole ring and ILE: 61, one electrostatic interaction between the benzene ring and HIS: 96, and two electrostatic interactions between nitro group and HIS: 96. It was noticed that the moieties that involved in the interactions with the selected proteins were pyrazole, indole, and *p*-nitrobenzene. It is worth mentioning that the *p*-nitrobenzene substituent played a great role in the binding to the active sites of the tested proteins.

**Table 3:** The values of Gibbs free energy (S) (Kcal/mol) and the types of interactions of compound **7f** with the active site of caspase 9, XIAP, P53 cancer mutant Y220C, and MDM2 proteins.

Compound 7f.	Proteins			
	Caspase 9	XIAP	P53 cancer mutant Y220C	MDM2
S (Kcal/mol)	− 21.8	− 16.5	− 20.1	− 24.7
Type of interactions	2 hydrogen bond	2 hydrogen bonds	2 hydrogen bond	1 hydrogen bond
	2 hydrophobic	2 hydrophobic	3 hydrophobic	3hydrophobic
	8 electrostatic	2 electrostatic	3 electrostatic	3 electrostatic
Standard ligand				
S (Kcal/mol)	− 33.7	− 24.9	− 15.8	− 33.9
RMSD	2	0.86	0.7	2

RMSD means root mean squared deviation.

## Discussion

The lead compound of our series is composed of indole, pyrazole, and acrylonitrile moieties. The diversity of the prepared compounds was based on the substituent (X) as mentioned before in the structure–activity relationship section. It was reported from the literature the promising biological activities of indole, pyrazole, and acrylonitrile which encouraged us to synthesize a series of compounds that combined all these moieties^51, 61, 62^. In our previous study^63^, It was found that 3-(thiophen-2-yl)pyrazol-4-yl)chalcone exerted a promising cytotoxic effect against HepG2 and A549 with IC_50_ values of 64 and 66.6 µM, respectively. Another example for pyrazolyl chalcone was 1-[3,5-dimethyl-1-(4-nitrophenyl)-1*H*-pyrazol-4-yl]-3-(*p*-tolyl) prop-2-en-1-one which showed promising anticancer activity against A549 and HepG2 with IC_50_ equalled 44.3 and 57.9 µg/cm^3^, respectively^64^. Regarding indolyl chalcones, Jumaah et al., found that (*E*)-3-(6-methoxy-1-methyl-1*H*-indol-3-yl)-1-(3,4,5-tri-methoxyphenyl)-prop-2-en-1-one, displayed high cytotoxic activities (IC_50_ = 19.18 µM) against MCF-7^65^. Kumar et al.^66^ suggested that the *trans* geometry of the molecule resulting from *α*-substitution may be responsible for the enhanced cytotoxic effects over unsubstituted counterparts. Lawrence et al.^67^ also studied the improved cytotoxic effects of the *α*-cyano substituent group on *α*,*β*-unsaturated enone system. The current study revealed that chalcone **7f** was the most effective compound against HCT116 (IC_50_ = 6.76 µg/mL) compared to 5-fluorouracil (IC_50_ = 77.15µg/mL). Regarding qPCR data, as depicted in Table 2, our findings showed that chalcone **7f** effectively up-regulated the apoptotic *BAX* gene with a fold change value of 3.662. Also, it effectively increased the expression level of the *P53* gene with a fold change of 5.435. On the other hand, chalcone **7f** strongly down-regulated gene expression of anti-apoptotic genes, *bcl2,* and *CDK4* with fold change values of 0.351 and 0.454, respectively. It was observed the high expression value of the tumour suppressor *p53* gene (5.435) relative to the control confirmed the promising cytotoxic effect of chalcone **7f** in the enhancement of the apoptotic death of colon cancer cells mediated *p53* mechanism. This current data was very compatible with our previous literature that tested derivatives similar to our current target chalcone against colon and breast carcinoma respectively^68, 69^. Most of the studies broadly emphasized the apoptotic effect of chalcone compounds as anticancer agents^70, 71^. Similarly, another study verified that chalcone derivatives are considered promising antitumor mediators as illustrated in earlier literature suggested that chalcones can stimulate apoptosis^72, 73^. The involvement of chalcone compounds in increasing the production of *caspases* has been evaluated previously by several studies using diverse carcinoma cells. For instance, Mohamed et al.^74^ agreed with this recent study and showed that heterocyclic cyanoacrylamide compounds such as *p*-fluorophenyl and* p*-phenolic compounds had an effect on *caspases* production in liver carcinoma cells (Hepg2) through increasing the activity of *caspases-3, -8,* and *-9*. Additional evidence for the interesting results outlined in this recent paper has also been reported by Syamet al.^70^ that showed apoptosis induction in breast cancer (MCF7) through stimulating the production of *caspase-7*, *caspase-8*, and *caspase-9* using synthesized chalcone derivatives. Also, it was reported the ability of chalcones to induce apoptosis in HCT116 cells^68^. Therefore, this was confirmed in the present study where our compound **7f** was capable of stimulating apoptosis in HCT116 cells by producing *caspases-3, -8,* and -*9* in a concentration-dependent manner as demonstrated in Figs. 6, 8, and 10, respectively. It is well established that *caspases* play a crucial role during the death of proteases as they are a family of cysteine-dependent aspartate-directed proteases. These caspases are classified into two main classes: upstream (initiator) caspases and downstream (effector) caspases. Upstream caspases involve *caspase-8* and *caspase-9* which can cleave inactive pro-forms of downstream caspases such as *caspase-3* and *caspase-7* that cleave proteins contributed in processes of cell^51, 75^. Furthermore, almost all studies approved that apoptosis can occur through two main pathways: the extrinsic (at the plasma membrane) and the intrinsic pathways (at the mitochondria)^76^. The activation of *caspases-3* and *-9* is elicited via the intrinsic pathway, whereas *caspase-3* and *-8* are activated through extrinsic pathway^74^. Therefore, we could conclude that our chalcone **7f** stimulated the apoptotic pathway through both intrinsic and extrinsic mechanisms. The ability to detect and identify intracellular molecules to characterize cell accumulations was performed using the flow cytometry technique. Chalcone **7f** efficiently induced cell cycle arrest at G1/S phase with percentage of 51.33% and 46.12%, respectively comparing to the untreated cells at the expense of (G2/M) phase. The DNA content in the mitotic phase G2/M was (2.55%) relative to the control sample (13.68%). In similar literature, as indicated by Guangcheng Wang et al.^77^, novel chalcones containing indole moiety clearly exhibited cell cycle arrest in G2/M in a dose-dependent manner compared to control cells in the Hepg2 cell line. At the G1 phase, CDK protein activity induced genome replication and stimulated the transition of the G1/S phase. CDKs initiated a positive feedback loop, which further enhanced CDK activities, and this forced the cell to be divided^78^. Herein, the cell cycle arrest at G1/S phase confirmed our result in the gene expression analysis section, where CDK4 was down-regulated. In addition, data obtained by flow cytometry clearly outlined that chalcone **7f** stimulated early apoptotic induction of colon cancer cells significantly to reach 21.38% compared to control cells (0.32%). The total apoptotic percentage in the sample treated with chalcone**7f** was a promising value (39.61%) relative to the untreated one (1.65%). Also, chalcone **7f** exerted certain stress on colon cancer-induced necrosis of its cells (6.82%) in comparison to the control (1.12%). In addition, chalcone **7f** completely degraded the genomic DNA in HCT116 cells supporting our flowcytometric results of apoptosis. As, DNA fragmentation is a late event of the apoptosis process. Molecular docking studies were done on caspase 9, XIAP, P53 cancer mutant Y220C and MDM2 proteins. Caspase 9 is an apoptotic initiator which its activation plays a critical role in the induction of apoptosis. It is an inactive monomer and its activation is triggered by dimerization^79^. This dimerization is induced via the interaction with a small molecule at the dimer interface triggering reorientation of the activation loop^79^. XIAP is a member of the apoptotic inhibitor protein family which inhibits apoptosis process through its inhibitory effect on caspase activity^80^. The tumor suppressor p53 is inactivated in many cancers by mutation and hence its reactivation with small molecules restores the apoptosis process^81^. MDM2 is a negative regulator of the expression of P53^82^. It was found from the molecular docking results that compound **7f** triggered the apoptosis through activating of caspase 9, reactivating p53 cancer mutant Y220C, and blocking XIAP and MDM2. These theoretical results were confirmed experimentally in the gene expression and ELISA sections. At the end of this current study, all these findings were surprising and suggested that our new chalcones especially compound **7f** might be an alternative promising therapeutic option, where it inhibited colorectal carcinoma through the induction of intrinsic and extrinsic apoptotic pathways of the apoptosis mediated p53 induction.

## Conclusion

Novel α-cyano-indolyl chalcones incorporating 1-phenyl-3-arylpyrazole were prepared and tested against two different cancer cell lines, lung and colorectal carcinoma. We outlined in this current paper that chalcone **7f**, a promising potential anticancer compound, induced human colorectal carcinoma cell death. It was found that chalcone **7f** regulated apoptosis by enhancing the expression of p53 and pro-apoptotic BAX genes, while it also depressed the production of several anti-apoptotic (bcl2 and CDK4) genes. **7f** strongly restricted cell growth in a dose-dependent manner as clearly indicated in the ELIZA assay regarding caspases-3,-8, and -9 productions. Chalcone **7f** caused apoptosis induction and G1/S cell cycle arrest in the HCT116 cell line. Chalcone **7f** completely degraded the genomic DNA of HCT116. A theoretical molecular docking study supported our experimental results in which chalcone **7f** interacted with caspase 9, XIAP, P53 mutant Y220C, and MDM2 with good binding energies, confirming the ability of **7f** to induce p53-dependent apoptosis mechanism (intrinsic and extrinsic pathways). Thus, we suggested that chalcone **7f** might be an alternative therapeutic option for colorectal carcinoma.

## Experimental

### Chemistry

Melting points were measured with using a Stuart melting point apparatus and were uncorrected. The IR spectra were recorded using an FTIR Bruker-vector 22 spectrophotometer as KBr pellets. The ^1^H and ^13^C NMR spectra were recorded in DMSO-*d*_*6*_ as a solvent with Varian Mercury VXR-300 NMR spectrometer operating at 300 MHz and 75 MHz, using TMS as an internal standard. Chemical shifts were reported as *δ* values in ppm. Mass spectra were recorded with a Shimadzu GCMS-QP-1000 EX mass spectrometer in EI (70 eV) model. The elemental analyses were performed at the Micro Analytical Centre, Cairo University.

#### General procedure for the synthesis of compounds 7a–7f

A mixture of cyanoacetyl-indole **3** (1 mmol) and appropriate 1-phenyl-3-arylpyrazole **6a–f** (1 mmol) was heated at reflux in absolute EtOH (10 mL) containing piperidine (0.2 mL) for 30 min. The crude product was collected and crystallized from EtOH/dioxane mixture.

#### (*E*)-3-(1,3-Diphenyl-1*H*-pyrazol-4-yl)-2-(1*H*-indole-3-carbonyl)acrylonitrile (7a)

Pale yellow crystals (90%). Mp: 250-252 °C. IR (KBr): ν_max_/cm^−1^ 3215 (NH), 2242 (CN), 1725 (CO). ^1^H NMR (300 MHz, DMSO-*d*_6_): *δ* 7.24–7.79 (m, 12H, Ar–*H*), 8.11 (s, 1H, vinyl-H), 8.16 (m, 2H, Ar–H), 8.52 (s, 1H, indole-H2), 9.25 (s, 1H, pyrazole-H), 12.22 (s, 1H, N*H*). ^13^C NMR (DMSO-*d*_6_): *δ* 108.9, 112.5, 113.8, 114.9, 118.5, 119.6, 121.5, 122.4, 123.6, 126.2, 128.0, 128.8, 128.9, 129.1, 129.2, 129.9, 130.9, 134.8, 136.5, 138.6, 143.6, 155.1, 180.1. MS (EI, 70 eV): 414 [M^+^], Anal. Calcd. for C_27_H_18_N_4_O: C, 78.24; H, 4.38; N, 13.52. Found: C, 78.35; H, 4.51; N, 13.73.

#### (*E*)-2-(1*H*-Indole-3-carbonyl)-3-(1-phenyl-3-(*p*-tolyl)-1*H*-pyrazol-4-yl)acrylonitrile (7b)

Pale yellow crystals (88%). Mp: 288-290 °C. IR (KBr): ν_max_/cm^−1^ 3180 (NH), 2252 (CN), 1710 (CO). ^1^H NMR (300 MHz, DMSO-*d*_6_): *δ* 2.38 (s, 3H, CH_3_), 7.24–7.96 (m, 11H, Ar–*H*), 8.10 (s, 1H, vinyl-H), 8.15 (m, 2H, Ar–H), 8.52 (s, 1H, indole-H2), 9.24 (s, 1H, pyrazole-H), 12.22 (s, 1H, N*H*). MS (EI, 70 eV): 428 [M^+^], Anal. Calcd. for C_28_H_20_N_4_O: C, 78.49; H, 4.70; N, 13.08. Found: C, 78.61; H, 4.88; N, 13.21.

#### (*E*)-2-(1*H*-Indole-3-carbonyl)-3-(3-(4-methoxyphenyl)-1-phenyl-1*H*-pyrazol-4-yl)acrylonitrile (7c)

Pale yellow crystals (78%). Mp: 252-254 °C. IR (KBr): ν_max_/cm^−1^ 3210 (NH), 2255 (CN), 1710 (CO). ^1^H NMR (300 MHz, DMSO-*d*_6_): *δ* 3.82 (s, 3H, OCH_3_), 7.09–7.97 (m, 11H, Ar–*H*), 8.16 (s, 1H, vinyl-H), 8.19 (m, 2H, Ar–H), 8.51 (s, 1H, indole-H2), 9.22 (s, 1H, pyrazole-H), 12.20 (s, 1H, N*H*). MS (EI, 70 eV): 444 [M^+^], Anal. Calcd. for C_28_H_20_N_4_O_2_: C, 75.66; H, 4.54; N, 12.60. Found: C, 75.76; H, 4.76; N, 12.69.

#### (*E*)-3-(3-(4-Hydroxyphenyl)-1-phenyl-1*H*-pyrazol-4-yl)-2-(1*H*-indole-3-carbonyl)acrylonitrile (7d)

Pale yellow crystals (83%). Mp: 288-290 °C. IR (KBr): ν_max_/cm^−1^ 3630 (NH), 3353 (NH), 2254 (CN), 1718 (CO). ^1^H NMR (300 MHz, DMSO-*d*_6_): *δ* 6.19 (d, 2H, Ar–H, *J* = 8.7 Hz), 7.26–7.27 (m, 2H, Ar–H), 7.45–7.60 (m, 5H, Ar–H), 7.91 (d, 2H, Ar–H, *J* = 7.5 Hz), 8.11 (s, 1H, vinyl-H), 8.23 (d, 2H, Ar–H, *J* = 8.7 Hz), 8.51 (s, 1H, indole-H2), 9.19 (s, 1H, pyrazole-H), 12.20 (br 2H, N*H* and OH). ^13^C NMR (DMSO-*d*_6_): *δ* 108.3, 112.5, 113.8, 114.7, 115.8, 118.6, 119.5, 121.5, 122.4, 123.6, 126.2, 127.9, 128.8, 129.9, 130.3, 134.7, 136.5, 138.7, 144.0, 155.4, 158.5, 180.2. MS (EI, 70 eV): 430 [M^+^], Anal. Calcd. for C_27_H_18_N_4_O_2_: C, 75.34; H, 4.21; N, 13.02. Found: C, 75.46; H, 4.39; N, 13.11.

#### (*E*)-3-(3-(4-Chlorophenyl)-1-phenyl-1*H*-pyrazol-4-yl)-2-(1*H*-indole-3-carbonyl)acrylonitrile (7e)

Pale yellow crystals (90%). Mp: 296-298 °C. IR (KBr): ν_max_/cm^−1^ 3182 (NH), 2252 (CN), 1708 (CO). ^1^H NMR (300 MHz, DMSO-*d*_6_): *δ* 7.09–8.02 (m, 13H, Ar–*H*), 8.21 (s, 1H, vinyl-H), 8.52 (s, 1H, indole-H2), 9.20 (s, 1H, pyrazole-H), 12.20 (br, 1H, N*H*). MS (EI, 70 eV): 448[M^+^], Anal. Calcd. for C_27_H_17_ClN_4_O: C, 72.24; H, 3.82; N, 12.48. Found: C, 72.36; H, 3.98; N, 12.61.

#### (*E*)-2-(1*H*-Indole-3-carbonyl)-3-(3-(4-nitrophenyl)-1-phenyl-1*H*-pyrazol-4-yl)acrylonitrile (7f)

Pale yellow crystals (89%). Mp: > 300 °C. IR (KBr): ν_max_/cm^−1^ 3150 (NH), 2252 (CN), 1722 (CO). ^1^H NMR (300 MHz, DMSO-*d*_6_): *δ* 7.24–7.28 (m, 2H, Ar–H), 7.48–7.65 (m, 4H, Ar–H), 7.95–7.98 (m, 5H, Ar–H), 8.01 (s, 1H, vinyl-H), 8.33 (d, 2H, Ar–H, *J* = 6.9 Hz), 8.52 (s, 1H, indole-H2), 9.27 (s, 1H, pyrazole-H), 12.22 (br, 1H, N*H*). ^13^C NMR (DMSO-*d*_6_): *δ* 110.5, 112.6, 113.8, 115.6, 118.2, 119.4, 119.8, 121.5, 122.5, 123.7, 124.2, 126.2, 128.3, 129.2, 129.9, 130.0, 135.2, 136.6, 137.5, 138.6, 142.8, 147.7, 152.5, 180.3. MS (EI, 70 eV): 459 [M^+^], Anal. Calcd. for C_27_H_17_N_5_O_3_: C, 70.58; H, 3.73; N, 15.24. Found: C, 70.67; H, 3.89; N, 15.46.

### Cytotoxic MTT assay

#### Source of cell lines (A549 and HCT116)

A549 and HCT116 cell lines were purchased from the Company for the production of vaccines, Sera, and drugs (VACSERA), Egypt.

MTT protocol was performed to study the viability of cells after treatment with the tested compounds. The studied cell lines (A549 and HCT116) were cultured and grown in RPMI‐1640 media, while HFB4 cells were reserved in DMEM‐F12 media. Both media were supplemented with 10% FBS, 1% L‐glutamine, and 1% antibiotic–antimycotic mixture. The cell lines were grown at conditions of 37 °C and 5% CO_2_. 10 × 10^3^ of A549, and HCT116 cells and the same count of normal melanocytes (HFB4) were cultured in a 96‐well plate. The selected cultured cells were kept at 37°C and 5% CO_2_ overnight. Then, the medium was discarded after 24 h of incubation, and 100 µL from different concentrations of the studied compounds dissolved in fresh medium without serum, with final concentrations of 100, 50, 25, 12.5, 6.25, 3.125, 1.56, and 0.78 µg/mL was added. The treated cells were then incubated for 48 h. After that, 50 µL of MTT was added into each well for 4 h for the formazan crystals to be formed. Finally, 200 µl of 10% sodium dodecyl sulphate (SDS) was added to each well, and plates were kept at room temperature overnight to allow the reaction to be stopped. The optical density at 575 nm was read using a microplate multi-well reader, and the percentage of cell viability was calculated. The dose–response curve was drawn to calculate the IC_50_ values for each compound using the prism GraphPad 5 program^83, 84^. 5 Fluorouracil, a commercial anticancer drug was used as a reference drug (positive control). Negative untreated cell control was used in this assay.

### RT-PCR

Total RNA was extracted from the compound **7f**-treated HCT116 cells and the untreated cells using the RNA Purification Kit GeneJET (Thermo scientific, ON, Canada) 0.1 µg of the extracted RNA was reverse transcribed into complementary DNA (cDNA) using High-Capacity cDNA Reverse Transcription Kit (Thermo Scientific, ON, Canada). qPCR was done using Brilliant SYBR Green qPCR master mix (Applied Biosystems, San Francisco, CA, USA) in One Step Plus Real-Time PCR System (Applied Biosystems, San Francisco, CA, USA). The total volume of the reaction was µL, 12.5 µL of SYBR green master mix, 2.5 µL of cDNA, 1 µL of each primer (*Bax, P53, Bcl2,* and *CDK4*) with 10 pmol/µL (Qiagen, CA, USA), and 9 µL of RNase free water. The thermal cycler was adjusted at an initial denaturation at 95 °C for 10 min, followed by 45 cycles at 95 °C for 15 s, 65 °C for 30 s, and 55 °C for 35 s. The relative gene expression was determined using the 2^−ΔΔCt^ equation after normalization to the expression of *β-actin* (housekeeping gene) (Qiagen, CA, USA)(10 pmol/µL)^85, 86^. Primer sequences used according to the literature as follows: *Bax*5′-ATGTTTTCTGACGGCAACTTC-3′(forward) and 5′-AGTCCAATGTCCAGCCCAT-3′ (reverse); for *Bcl2* 5′-ATGTGTGTGGAGACCGTCAA-3'(forward) and 5'′GCCGTACAGTTCCACAAAGG-3′ (reverse); for *p53*5′-ATGTTTTGCCAACTGGCCAAG-3′ (forward) 5′- TGAGCAGCGCTCATGGTG-3′ (reverse); for *CDK4* 5′-TCGAAAGCCTCTCTTCTGTG-3`(forward) and 5′-TACATCTCGAGGCCAGTCAT-3′ (reverse); for *β-actin* 5′-GTGACATCCACACCCAGAGG-3′ (forward) and 5′ ACAGGATGTCAAAACTGCCC-3′ (reverse).

### ELISA assay

The quantitative measurements of Human *caspases-3, -8,* and -*9* concentrations in HCT116 cell culture lysates were performed using ELISA assay. The protocols were followed up based on the manuscript instructions described in the following kits; Invitrogen human *Caspase-3* Elisa Kit, Catalog # KHO1091, DRG® human *Caspase-8* ELISA Kit, Catalog # **(**EIA-4863**)**, and DRG® human *Caspase-9* ELISA Kit, Catalog # (EIA-4860)respectively. In short, the procedure described in the above protocols was as instructed; all reagents, standards, and samples were prepared. Then, 100 μL of standard or samples were added to each well and incubated at room temperature for 2 h. Afterward, 100 μL of the prepared antibody (anti-rabbit-IgG-HRP) was added to all wells for 1 h at room temperature. Subsequently, 100 μL of the prepared TMB (tetramethyl-benzidine) substrate solution was added to all wells and incubated for 15 min. Finally, 50 μL of stop solution was added to all wells to completely inactivate the effect of the enzyme. All readings were measured immediately at a wavelength of 450 nm. From the readings of the standards, a standard curve was plotted using curve fitting software. Then, the concentrations for unknown samples and controls were measured from the standard curve^87^.

### Flow cytometric analysis of cell cycle

2 × 10^6^ of HCT116 cells were inoculated in 60 mm Petri dishes for 24 h and then treated with compound **7f** at its IC_50_ concentration for 48 h. In this study, non-treated HCT116 cells were used as a negative control. After the incubation period of 48 h, HCT116 treated cells were centrifuged at 1,200 rpm at 4 °C for 10 min. After disposing of the supernatant, the cell pellet was suspended in phosphate-buffered saline (PBS) and then centrifuged at 1200 rpm for 10 min. 70% cold ethanol was added overnight to allow the fixation of the cell pellet. After the step of centrifugation, the propidium iodide (PI) mixture was added to the cell pellet for 30 min at room temperature in the darkness. Subsequently, cells were subjected to Epics XL-MCL flow cytometer (BeckmanCoulter, Miami, FL) for analysis of DNA content^88^. The percentage of cells in different phases of the cell cycle was analyzed by Multicycle software (Phoenix Flow Systems, San Diego, CA).

### Flow cytometric analysis of apoptosis

The percentage of apoptotic cells was detected using Annexin V-FITC kit catalogue number (#K101-25). Briefly, 5 × 10^5^ of HCT116 cells were collected by centrifugation. Then, the cell pellet was suspended in 500 µL of 1X Binding Buffer. Afterward, 5 µL of Annexin V-FITC and 5 µL of propidium iodide were added to cells and kept at room temperature for 5 min in the dark^89–91^. Finally, the percentage of the apoptotic cells was detected by flow cytometry using the FITC signal detector(usually FL-1) and PI staining by the phycoerythrin emission signal detector (usually FL-2).

### DNA fragmentation

The DNA gel electrophoresis laddering assay in the HCT116 cell line was performed according to the instructions of Yawata^87^ with some modifications. Briefly, after 48 h of exposure of the HCT116 cancer cell line to compound **7f** in Petri dishes (60 × 15 mm, Greiner), the cells were suspended in 1 ml of medium and then centrifuged at 900 rpm for 10 min. The genomic DNA was extracted as demonstrated in Yawata^87^. About 1 × 10^6^ cells of HCT116 were treated with the IC_50_ value of compound **7f**. Then, the treated cells were collected *via* trypsinization and washed with Dulbecco’s Phosphate Buffered Saline. Afterwards, the cells were treated with the lysis buffer (5 mM ethylenediaminetetraacetic acid (EDTA), 0.5% Triton X-100, 10 mM Tris (pH 7.4), and 150 mM NaCl) for 35 min on ice. Then, the lysates were centrifuged at 10.000xg for 20 min. The degraded DNA was extracted from the supernatant with an equal volume of isoamyl alcohol: chloroform: neutral phenol mixture (1:24:25). At last, the fragmented DNA percentage was detected by performing gel electrophoresis using 2% agarose gel containing 0.1 μg/ml ethidium bromide (Supplementary File 1).

### Docking study

A molecular docking study was performed to demonstrate the interactions between compound 7f and the active site of Caspase 9, X-linked inhibitor of apoptosis protein (XIAP), p53 cancer mutant Y220C, and Mouse double minute 2 homolog (MDM2) proteins. The most promising compound 7f was designed and drawn in Molecular Operating Environment (MOE) modelling program 2009.10 version. Then, the energy of the studied compound was minimized by using MMFF94x as a force field, and partial charges were added. Afterward, compound 7f was saved in the (MOE) program in mdb format in 3D form, which was used for the next step of docking with the protein. The proteins were downloaded from the site of Protein Data Bank (www.rcsb.org, with PDB code: 1jxq, 3eyl, 5o1h, and 4wt2 respectively). The proteins with their co-crystallized standard ligands ((benzoxycarbonyl-Val-Ala-Asp-fluoromethyl ketone), ((3S,6S,7R,9aS)-6-{[(2S)-2aminobutanoyl]amino}-7-(2-aminoethyl)-*N*-(diphenylmethyl)-5-oxooctahydro-1H-pyrrolo[1,2-a]azepine-3-carboxamide), (3-iodanyl-2-oxidanyl-5-propylsulfanyl-4-pyrrol-1-yl-benzoic acid**)**, and (4-({[(3R,5R,6S)-1-[(1S)-2-(tert-butylsulfonyl)-1-cyclopropylethyl]-6-(4-chloro-3-fluorophenyl)-5-(3-chlorophenyl)-3-methyl-2-oxopiperidin-3-yl]acetyl}amino)-2-methoxybenzoic acid) respectively, were prepared for docking as followed, water molecules were removed and hydrogen atoms were added by protonate 3D to the protein structure. After the docking step was finished, the interactions were saved as a Pdb file and visualized through the BIOVIA Discovery Studio V6.1.0.15350 program, where the target compound appeared to fit into the active domain of the studied proteins in 2D and 3D forms^83, 92^.

### Supplementary Information

Supplementary Figures.

## Data Availability

Data available at request (Dr. Magda F. Mohamed, email:magdafikry85@yahoo.com, ismail_shafy@yahoo.com).
