# Supplementary figures and images for: Novel 3-(pyrazol-4-yl)-2-(1H-indole-3-carbonyl)acrylonitrile derivatives induce intrinsic and extrinsic apoptotic death mediated P53 in HCT116 colon carcinoma
Source: Sci Rep. 2023 Dec 15;13:22486. doi: 10.1038/s41598-023-48494-7 (PMC10728220; doi:10.1038/s41598-023-48494-7)

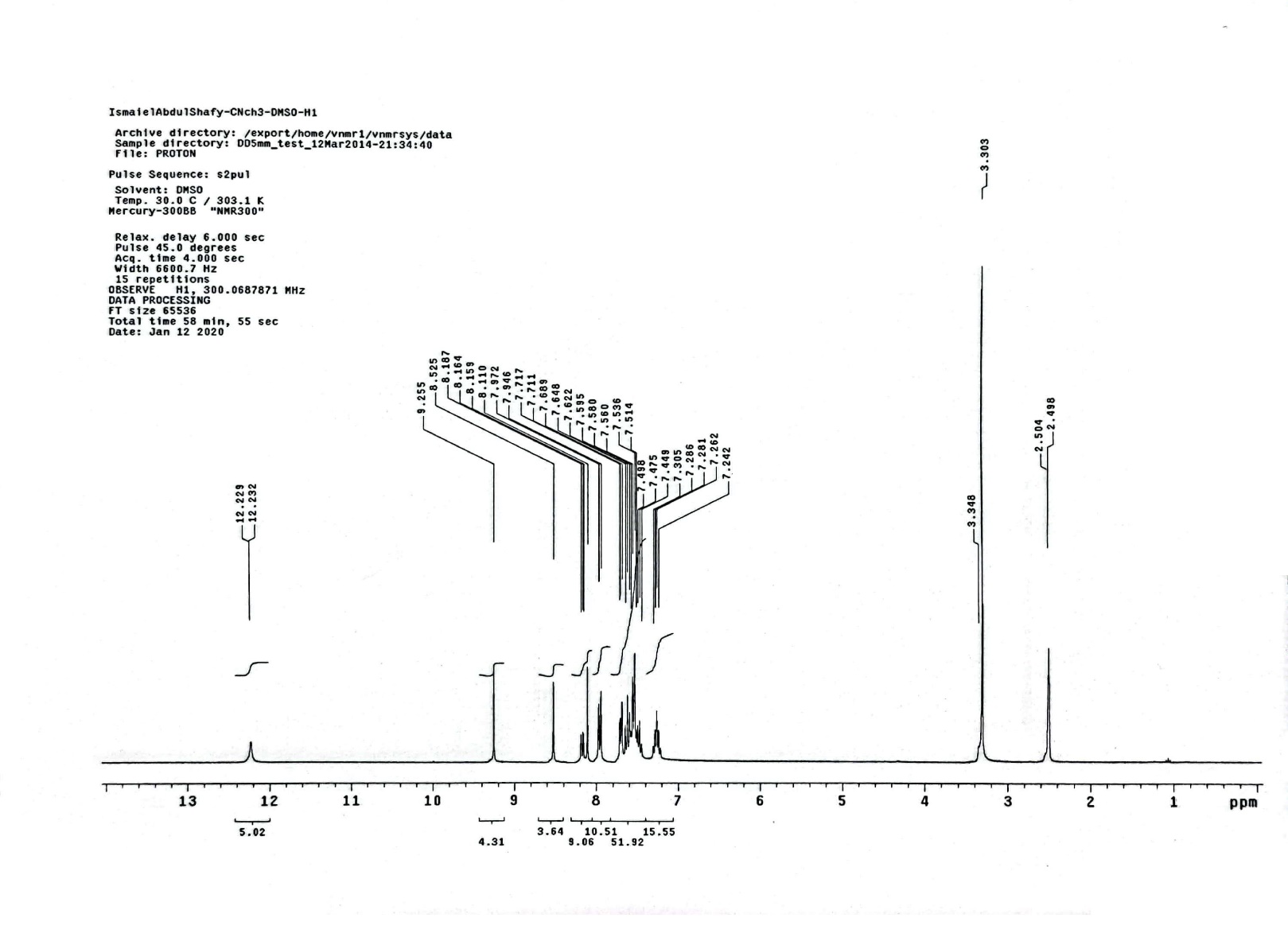


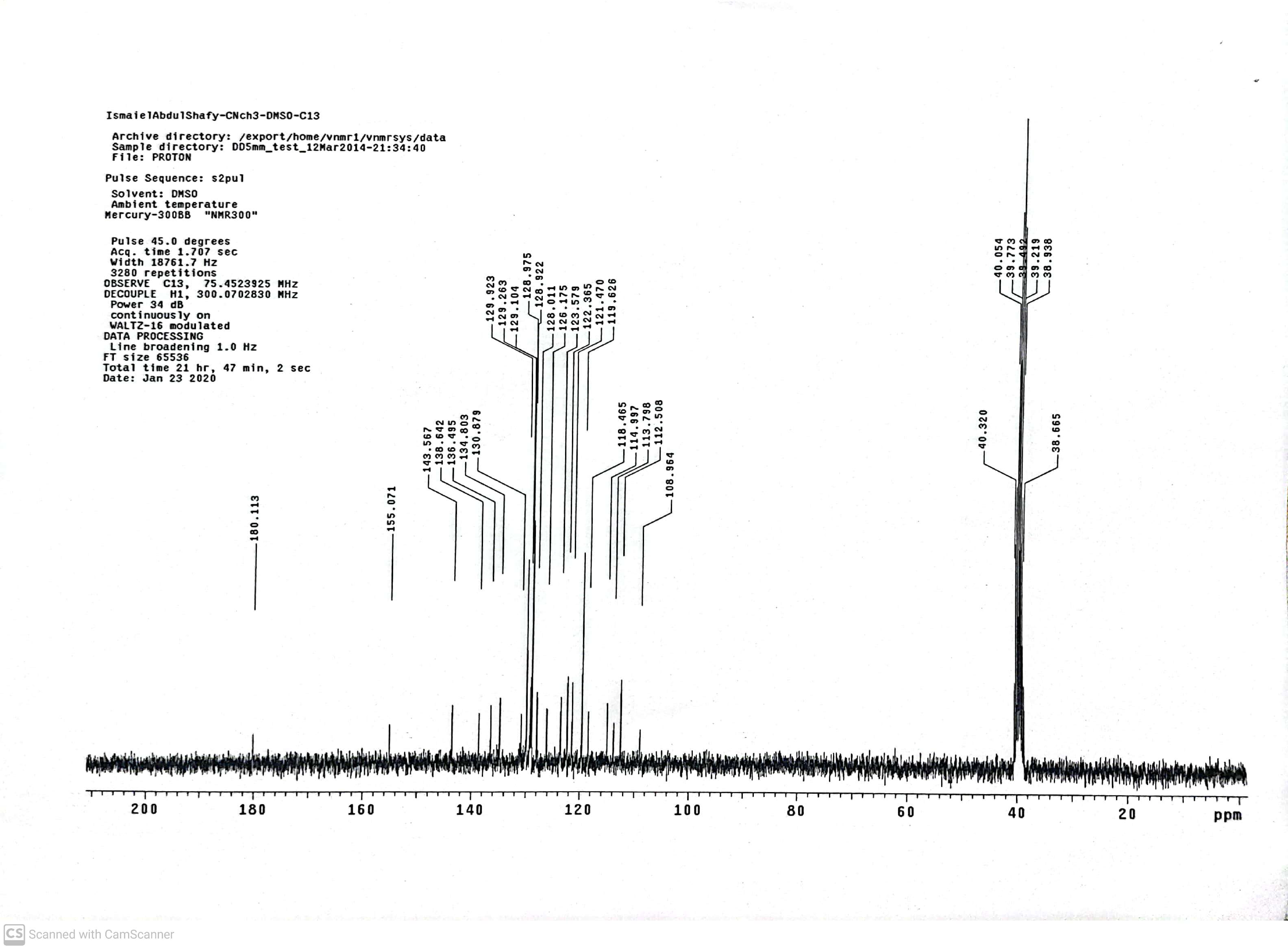


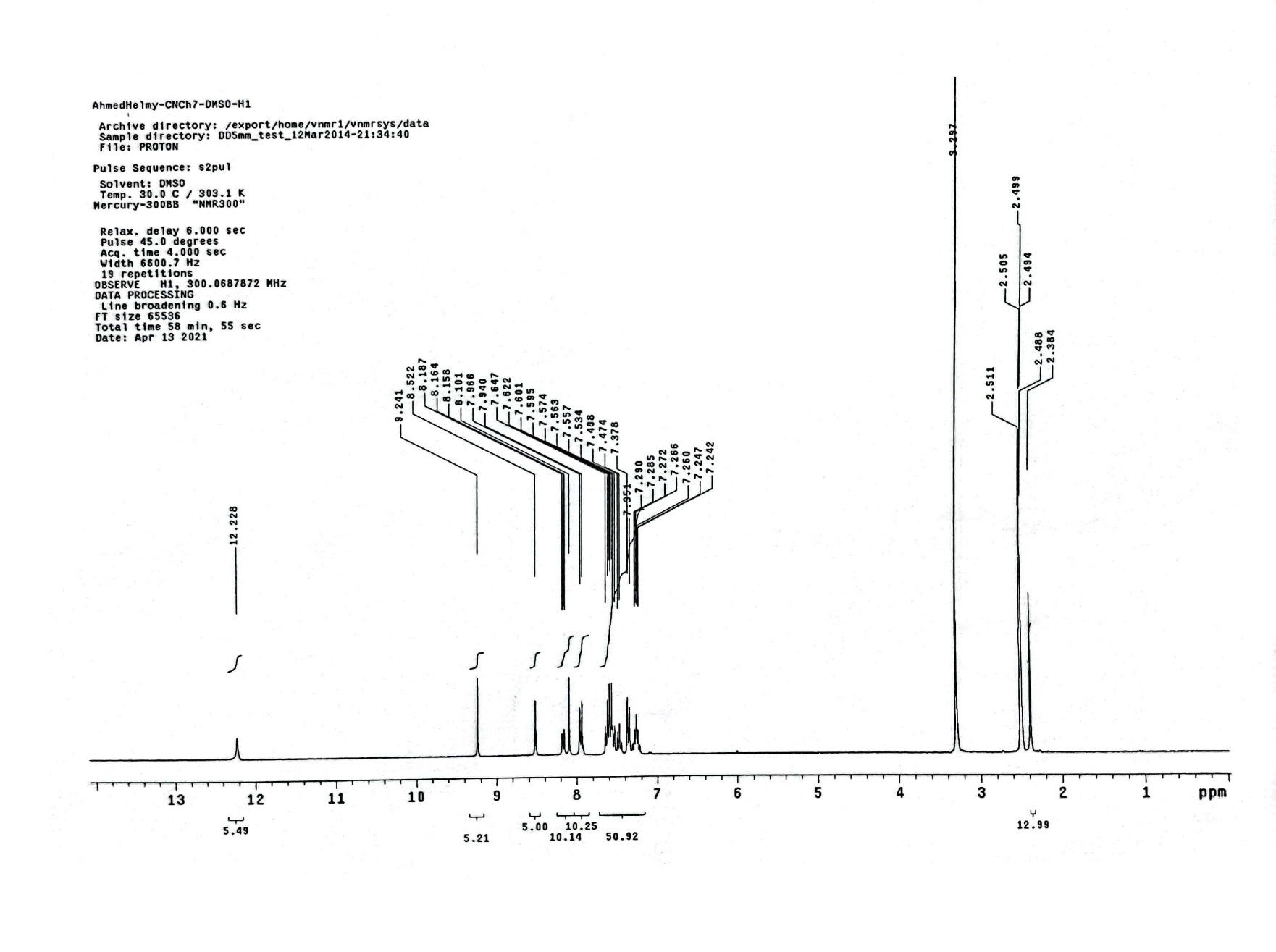


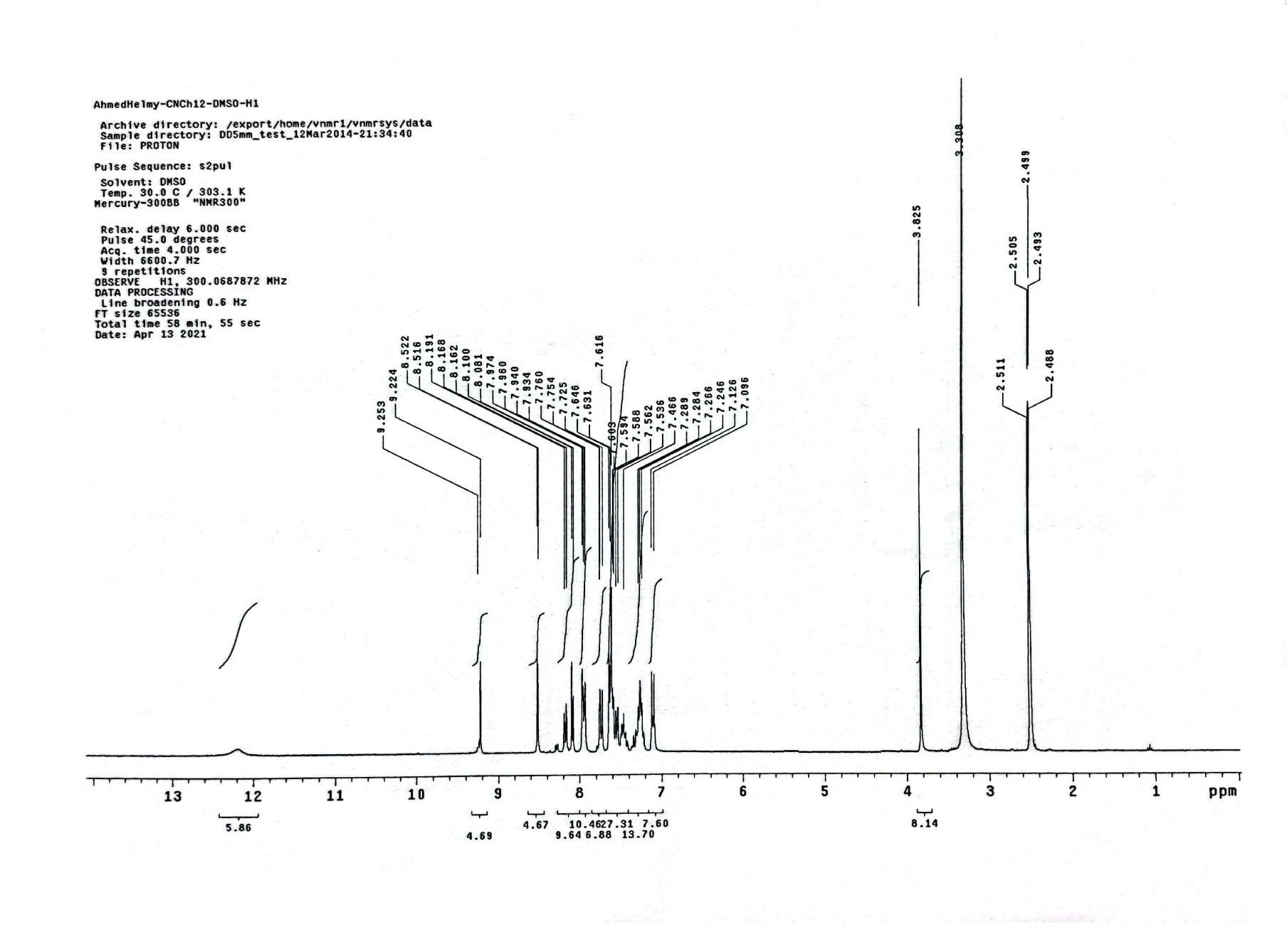


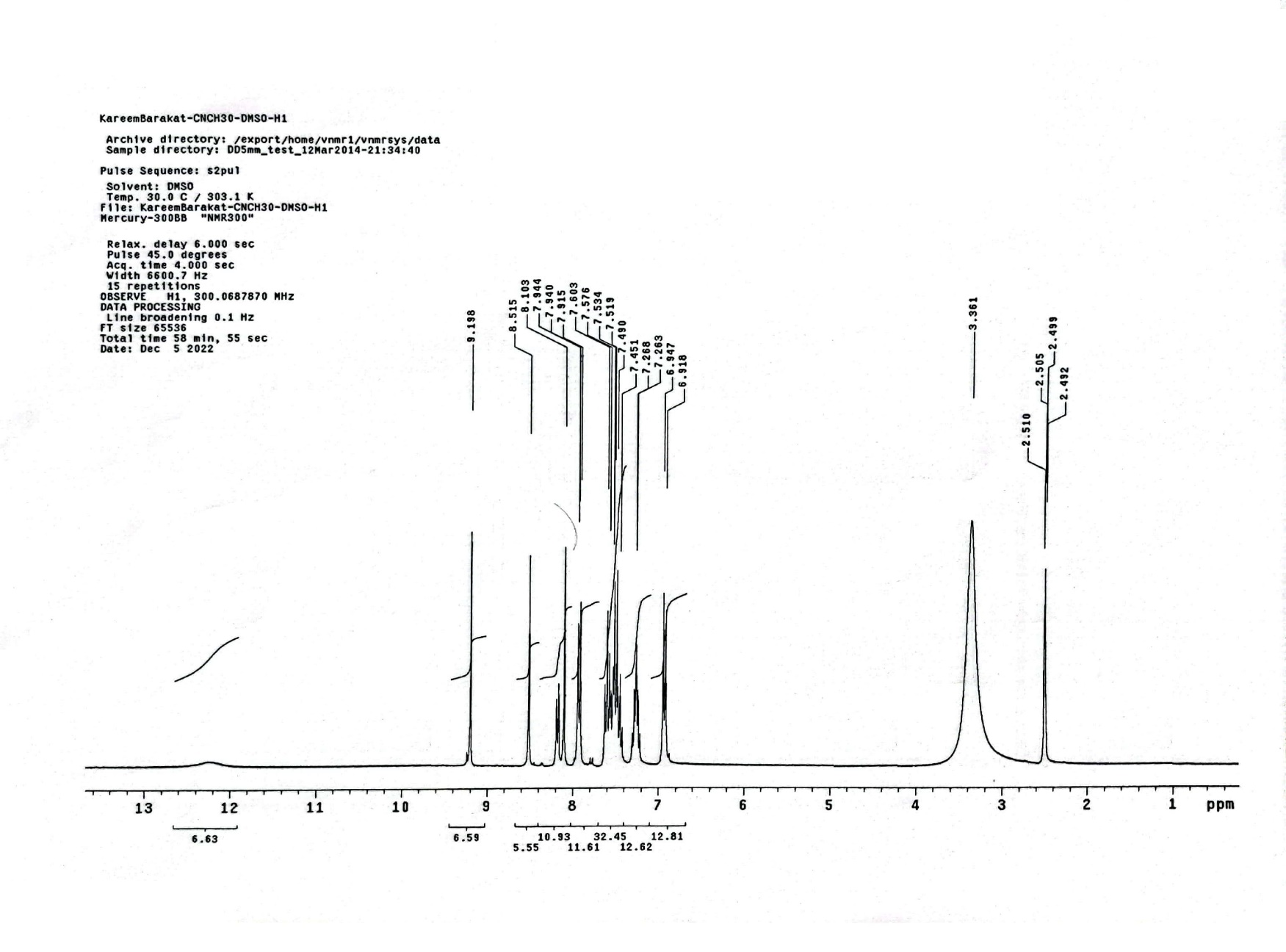


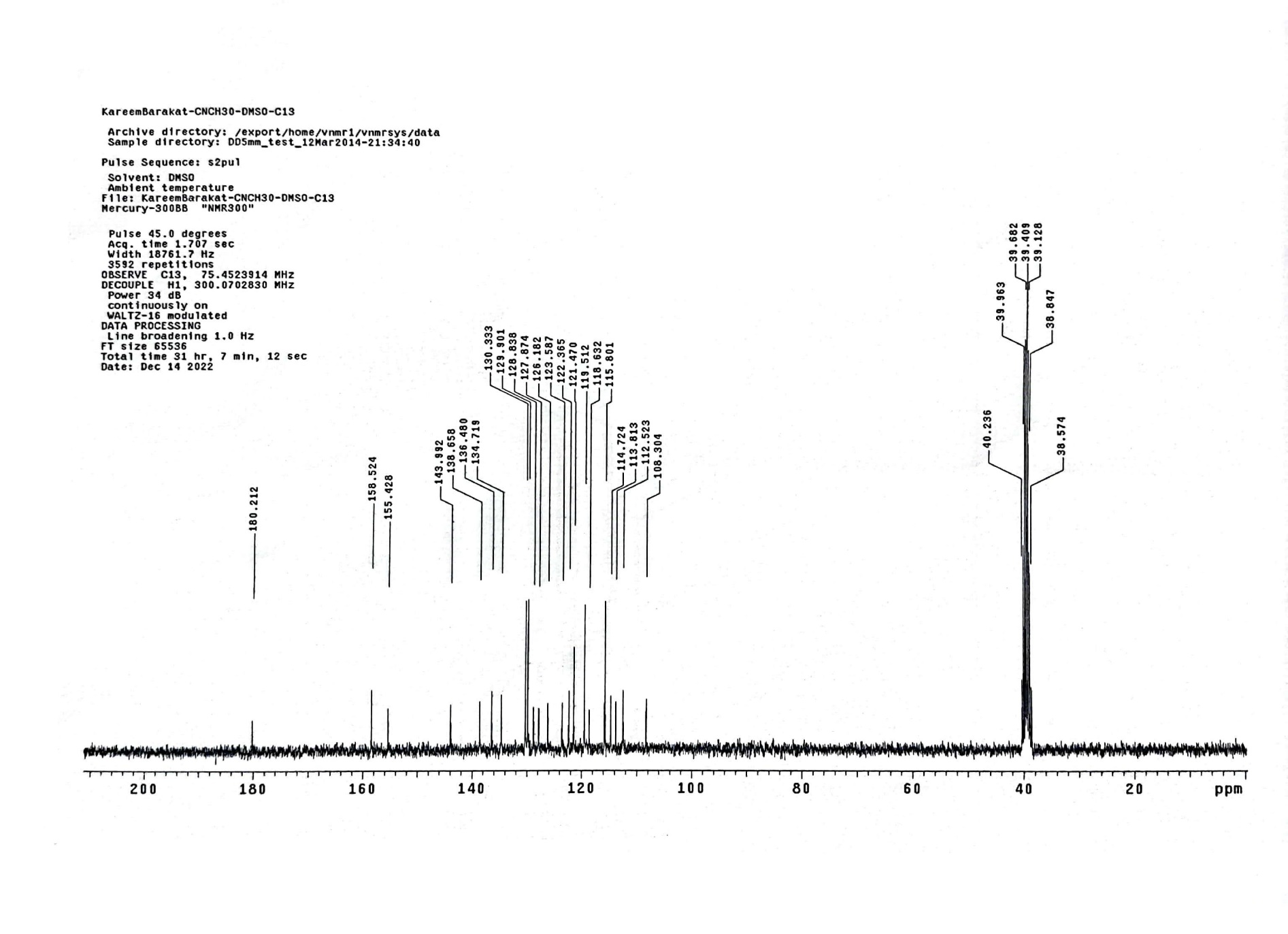


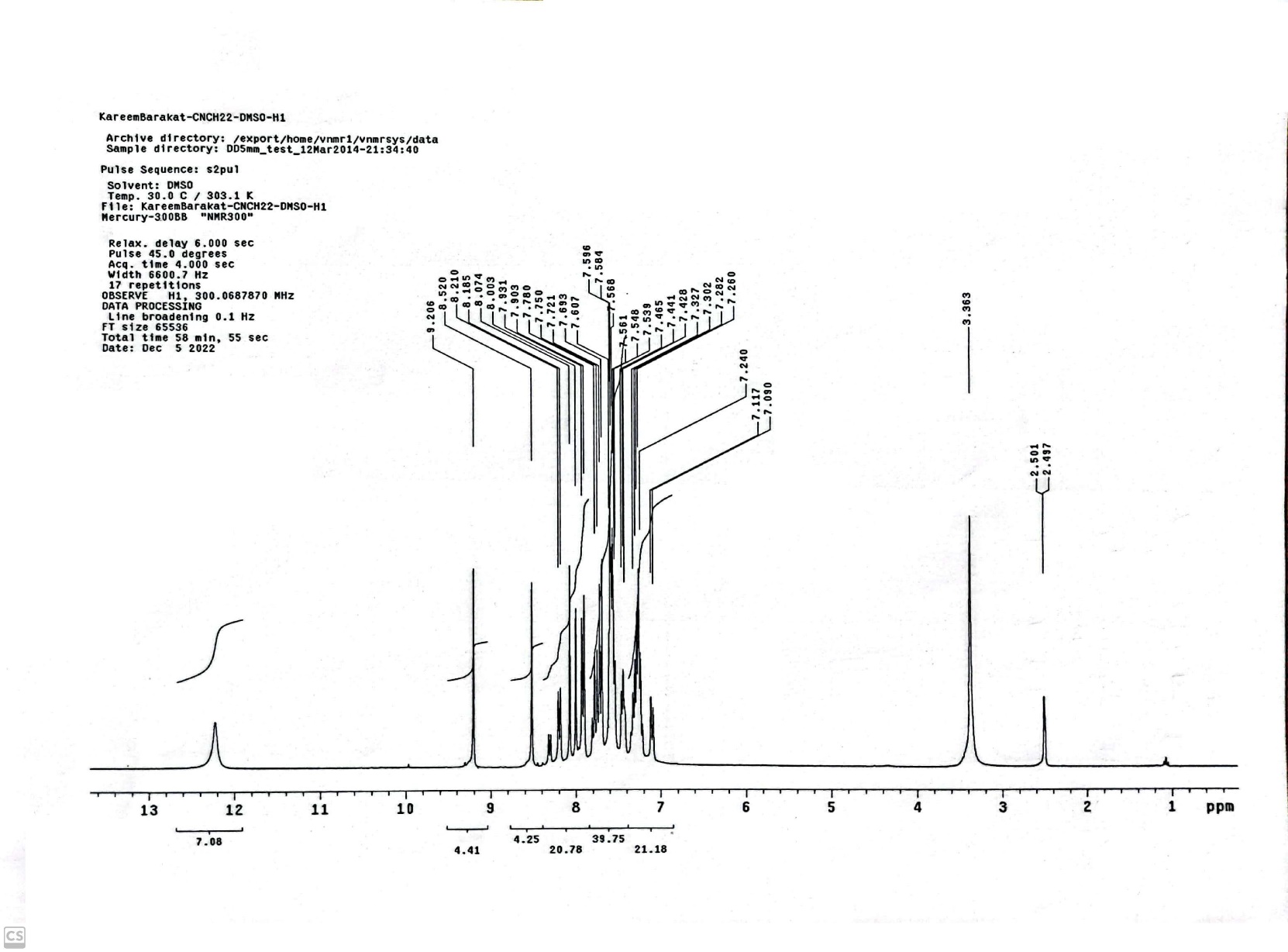


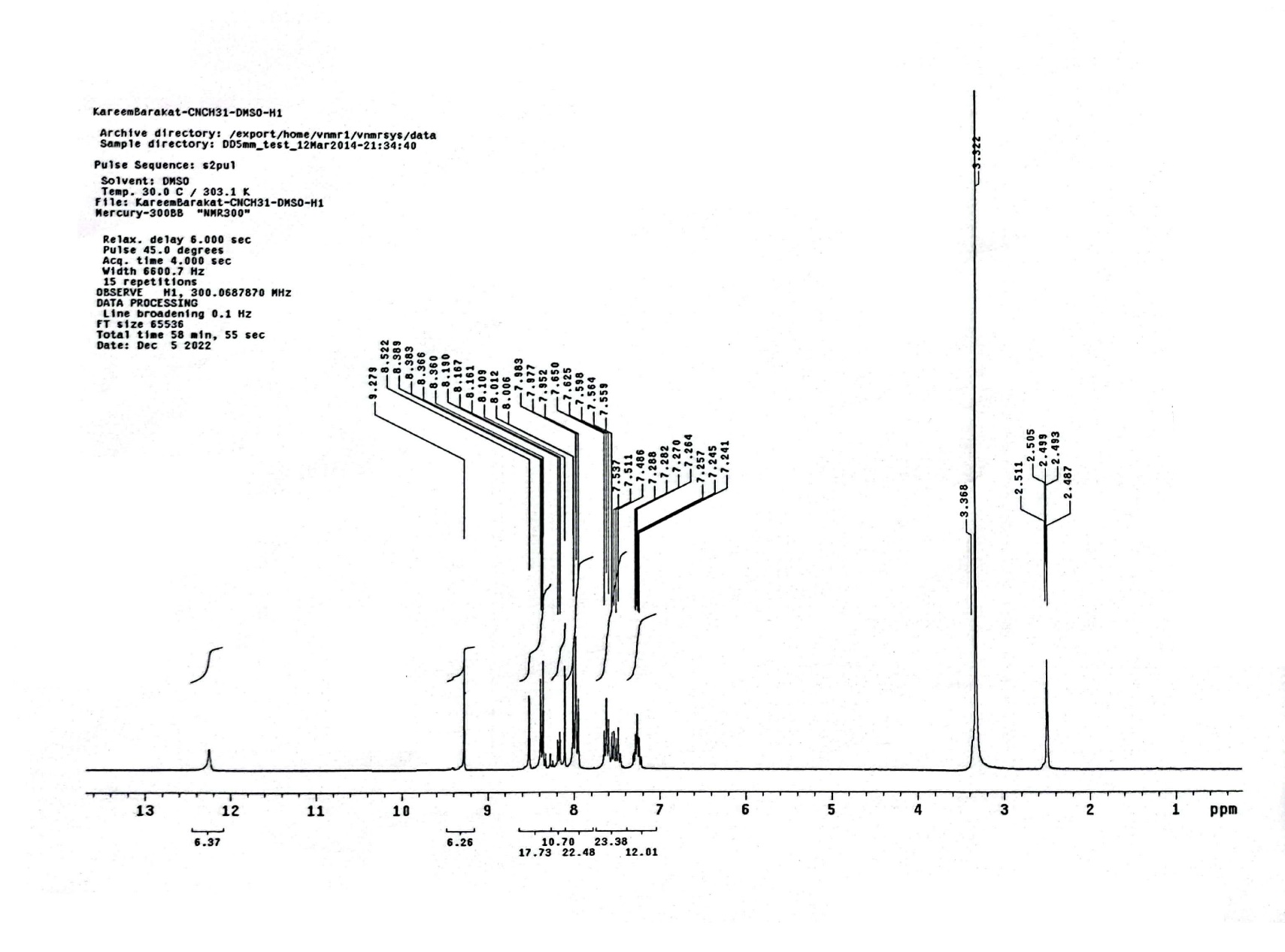


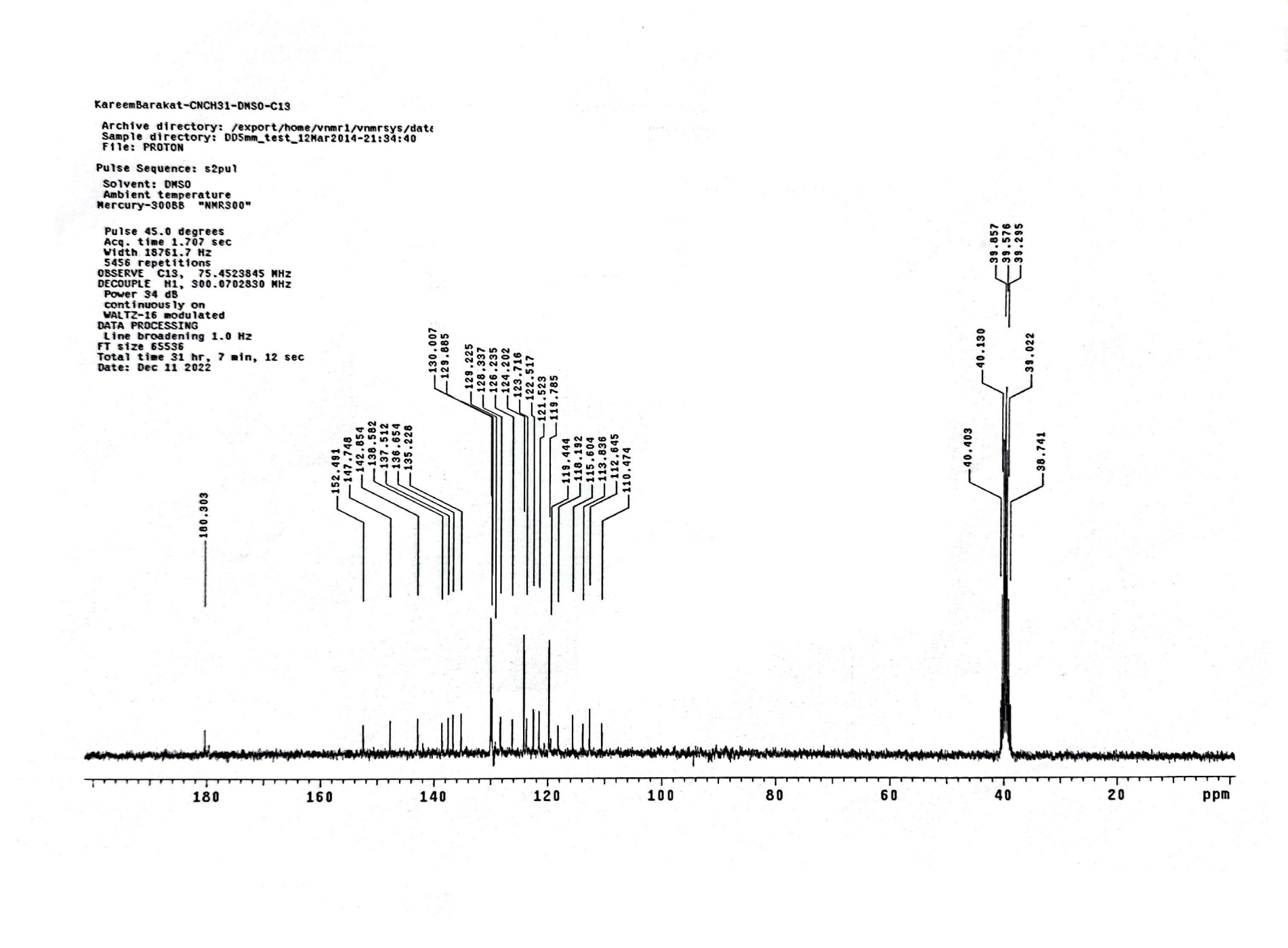

Supplement: Supplementary file 1 — Supplementary Figures. [file 41598_2023_48494_MOESM1_ESM.docx]
